# Anillin governs mitotic rounding during early epidermal development

**DOI:** 10.1186/s12915-022-01345-9

**Published:** 2022-06-16

**Authors:** Adnan Mahly, Krishnanand Padmanabhan, Arad Soffer, Jonathan Cohen, Jana Omar, Ronit Sagi-Eisenberg, Chen Luxenburg

**Affiliations:** Department of Cell and Developmental Biology, Sackler Faculty of Medicine, Tel Aviv University, P.O. Box 39040, 69978 Tel Aviv, Israel

**Keywords:** Anillin, Actin, Mitotic rounding, Cytokinesis, Adhesion, Morphogenesis, Development, Skin, Epidermis

## Abstract

**Background:**

The establishment of tissue architecture requires coordination between distinct processes including basement membrane assembly, cell adhesion, and polarity; however, the underlying mechanisms remain poorly understood. The actin cytoskeleton is ideally situated to orchestrate tissue morphogenesis due to its roles in mechanical, structural, and regulatory processes. However, the function of many pivotal actin-binding proteins in mammalian development is poorly understood.

**Results:**

Here, we identify a crucial role for anillin (ANLN), an actin-binding protein, in orchestrating epidermal morphogenesis. In utero RNAi-mediated silencing of *Anln* in mouse embryos disrupted epidermal architecture marked by adhesion, polarity, and basement membrane defects. Unexpectedly, these defects cannot explain the profoundly perturbed epidermis of *Anln*-depleted embryos. Indeed, even before these defects emerge, *Anln*-depleted epidermis exhibits abnormalities in mitotic rounding and its associated processes: chromosome segregation, spindle orientation, and mitotic progression, though not in cytokinesis that was disrupted only in *Anln*-depleted cultured keratinocytes. We further show that ANLN localizes to the cell cortex during mitotic rounding, where it regulates the distribution of active RhoA and the levels, activity, and structural organization of the cortical actomyosin proteins.

**Conclusions:**

Our results demonstrate that ANLN is a major regulator of epidermal morphogenesis and identify a novel role for ANLN in mitotic rounding, a near-universal process that governs cell shape, fate, and tissue morphogenesis.

**Supplementary Information:**

The online version contains supplementary material available at 10.1186/s12915-022-01345-9.

## Background

During development, cell shape, migration, proliferation, and differentiation must be tightly synchronized to create tissues with defined structure and function. In dynamic tissues such as the mammalian epidermis, which maintains its barrier function while cells continuously proliferate at the basal layer, differentiate in the suprabasal layers, and slough from the surface of the body, this synchronization must be maintained throughout life [review in [1–4]].

The actin cytoskeleton is ideally situated to orchestrate tissue development and homeostasis due to its roles in mechanical, structural, and regulatory processes [5–7]. In the developing epidermis, actin and its binding proteins are essential for basement membrane (BM) organization, cell adhesion, cell polarity, cell shape, spindle orientation, and cell delamination, all of which are necessary for the establishment of the epidermis’s structure and function [8–17]. In line, mutations in actin regulators were shown to play a role in common skin diseases that hinder epidermal structure and function such as psoriasis [18–20] and cancer [21, 22]. However, despite their essential roles, the function of many pivotal actin-binding proteins in the mammalian epidermis remains poorly understood.

Encoded by *Anln*, the protein anillin was isolated more than 30 years ago from *Drosophila* embryos thanks to its ability to bind filamentous (F-) actin [23, 24]. Later on, ANLN orthologs were identified in many eukaryotic organisms, and ANLN was shown to function as an actin-bundling and actin-scaffolding protein that directly interacts with a number of actin regulators such as the motor protein Myosin II, the formin DIAPH3, and the small GTPase RhoA [25–30] [31]. ANLN has a well-established role in cell division, with its loss of function resulting in cytokinesis failure and multinucleation of many cell types [review in [27, 28, 32, 33]]. In non-mitotic epithelial cells, ANLN was shown to be essential for cell-cell adhesion in a variety of cultured human epithelial cells [34] and in the developing Xenopus [35, 36]. ANLN also plays a role in the migration of cultured human epithelial cells [37] and in ventral enclosure in the developing *C. elegans* [38].

In humans, missense mutations in ANLN hinder the function of podocytes, epithelial cells of the glomerulus, causing focal segmental glomerulosclerosis (FSGS) and kidney failure [39]. Moreover, abnormal levels of ANLN were reported in many types of epithelial cancers, including breast, colorectal, pancreatic, lung, and head and neck squamous cell carcinomas [40–43]. While these observations strongly suggest that ANLN activity is essential for epithelial health, to our knowledge, its functions in physiologically relevant mammalian epithelia have never been explored.

Here, we investigated the role of ANLN in the developing mouse epidermis by RNAi-mediated gene silencing in utero. We show that ANLN is a major regulator of epidermal morphogenesis. ANLN-depleted epidermis exhibits early defects in mitotic rounding and later defects in cell-cell adhesion, polarity, and BM organization. ANLN orchestrates all these diverse functions by regulating cortical actomyosin’s levels, activity, and structural organization. These observations delineate a novel function for ANLN in mitotic rounding a highly conserved process that orchestrates cell shape, fate, and tissue morphogenesis.

## Results

### *Anln* loss-of-function hinders epidermal integrity

**Fig. 1:**
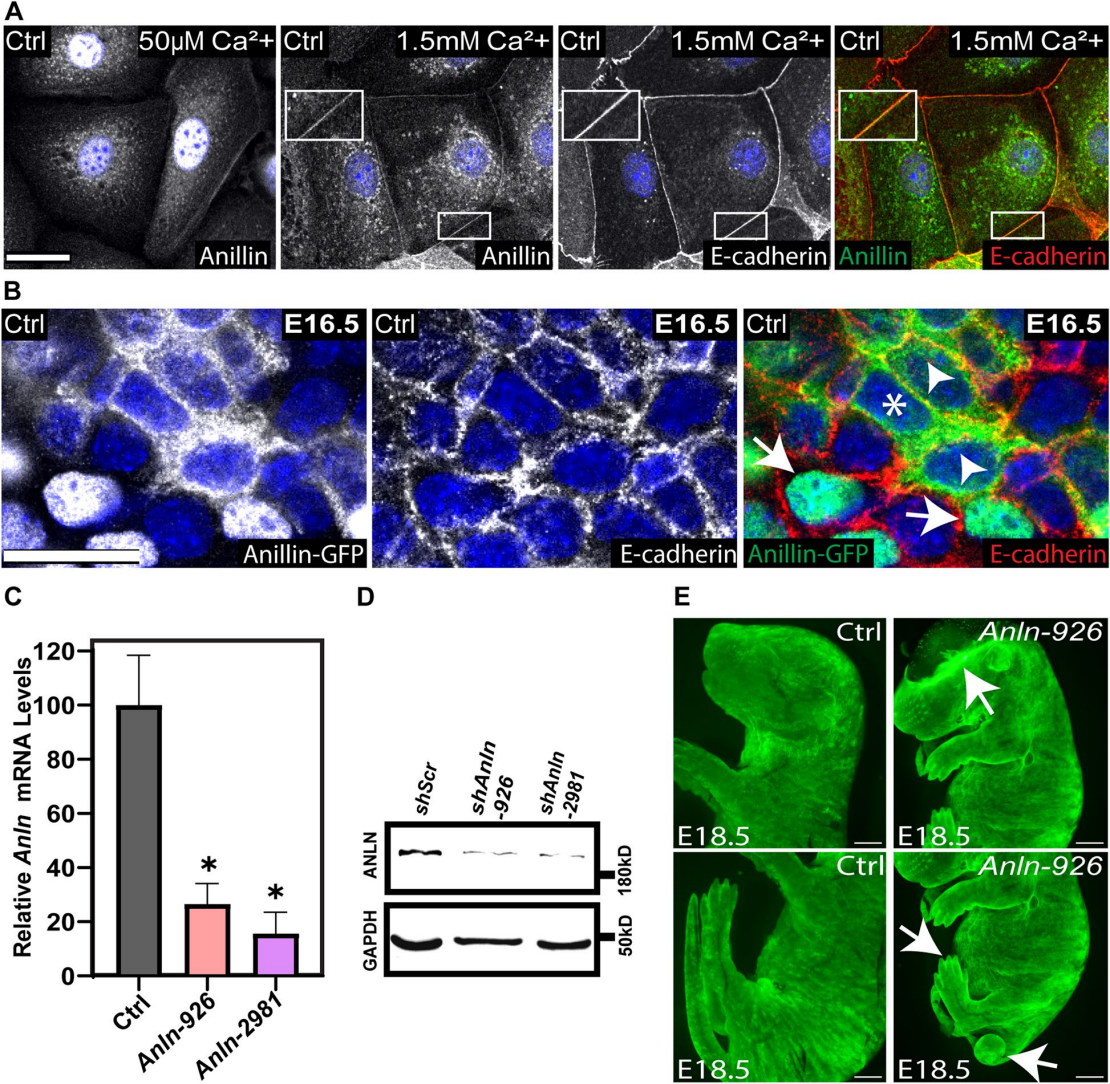
*Anln* depletion in the developing epidermis disrupts tissue integrity. **A** Wild-type keratinocytes were cultured in media containing 50μM or 1.5mM calcium, fixed, and labeled for ANLN and E-cadherin. Scale bar = 20 μm. **B** Whole-mount immunofluorescence images of *Anln-GFP*-transduced E16.5 embryos immunolabeled for E-cadherin and imaged at the middle of the basal layer. Arrows denote nuclear localization, arrow heads denote cytoplasmatic, and asterisk denotes cortical localization. Scale bar = 20 μm. **C** Quantitative PCR analysis of *Anln* mRNA in primary mouse keratinocytes transduced with scrambled shRNA (Ctrl) or one of two *Anln*-specific shRNAs (926 and 2981). Data are the mean ± SD of four preparations. *, *P* < 0.0001 (Scr vs. *Anln*-926); *, *P* < 0.0001 (Scr vs. *Anln*-2981) by unpaired *t*-test. **D** Western blot analyses of primary mouse keratinocytes transduced with Scr, *Anln*-926, or *Anln*-2981 shRNAs. Blots were probed with antibodies to ANLN and GAPDH (loading control). **E** Stereomicroscopic images of E18.5 embryos infected on E9 with *shScr;H2B-GFP* (Ctrl) or *shAnln-926;H2B-GFP* lentiviruses. Arrows denote defects in the head, tail, and limbs. Scale bar = 2 mm

In several experimental systems, ANLN was shown to shuttle between the nucleus and the cortex [23, 36, 44]. To understand the function of ANLN, we first examined its localization in primary mouse keratinocytes (1^0^MKs). In a low-calcium medium (50 μM), in which 1^0^MKs do not form cell-cell junctions, an immunofluorescence (IF) analysis detected ANLN in the nucleus, cytoplasm, and the cortex of the cells. When cultured in a high-calcium medium (1.5 mM), conditions that allow 1^0^MKs to form cell-cell junctions, ANLN was also detected at the junctions, where it co-localized with the adhesion receptor E-cadherin (Fig. 1A). To determine the localization of ANLN in the developing epidermis, we injected lentiviruses harboring GFP-tagged *Anln* [45] into the embryonic sacs of embryonic day (E)9 CD1 mouse embryos. Analysis of the basal layer from the dorsal skin of E16.5 embryos showed that ANLN-GFP localized to the nucleus, cytoplasm, or cell periphery, where it co-localized with E-cadherin (Fig. 1B).

To study the function of ANLN in skin development, we first identified two *Anln*-specific short hairpin RNAs (shRNAs), termed *Anln-926* and *Anln-2981*, that depleted *Anln* mRNA levels in primary mouse keratinocytes by 73.4 ± 7.6% and 84.4 ± 7.9%, respectively (Fig. 1C), and reduced ANLN protein expression by comparable levels (Fig. 1D). Next, we injected the embryonic sacs of E9 wild-type mouse embryos in utero with lentiviruses expressing *Anln-926* or scrambled shRNA (control) together with a GFP-tagged histone 2B reporter (H2B-GFP), to identify transduced cells [46]. A fluorescence stereomicroscopy examination of the embryos at E18.5 revealed profound defects in the embryos’ appearance. Namely, we detected curved tails in ~60% of the *shAnln*-transduced embryos and short limbs and paucity of skin in the head region in ~40% of the *shAnln*-transduced embryos, defects that were not detected in control, *shScr*-transduced embryos (Fig. 1E).

### Adherens junction defects are followed by polarity and basement membrane defects in *Anln*-depleted epidermis

As our results suggest that ANLN loss-of-function severely disrupts epidermal morphogenesis, we sought to analyze how this loss-of-function impacts factors vital for epidermal morphogenesis, namely, cell adhesion, cell polarity, and BM organization. We focused on the period from E14.5, when the first suprabasal layer can be detected, to E18.5, when the epidermal barrier is functional [47, 48].

**Fig. 2:**
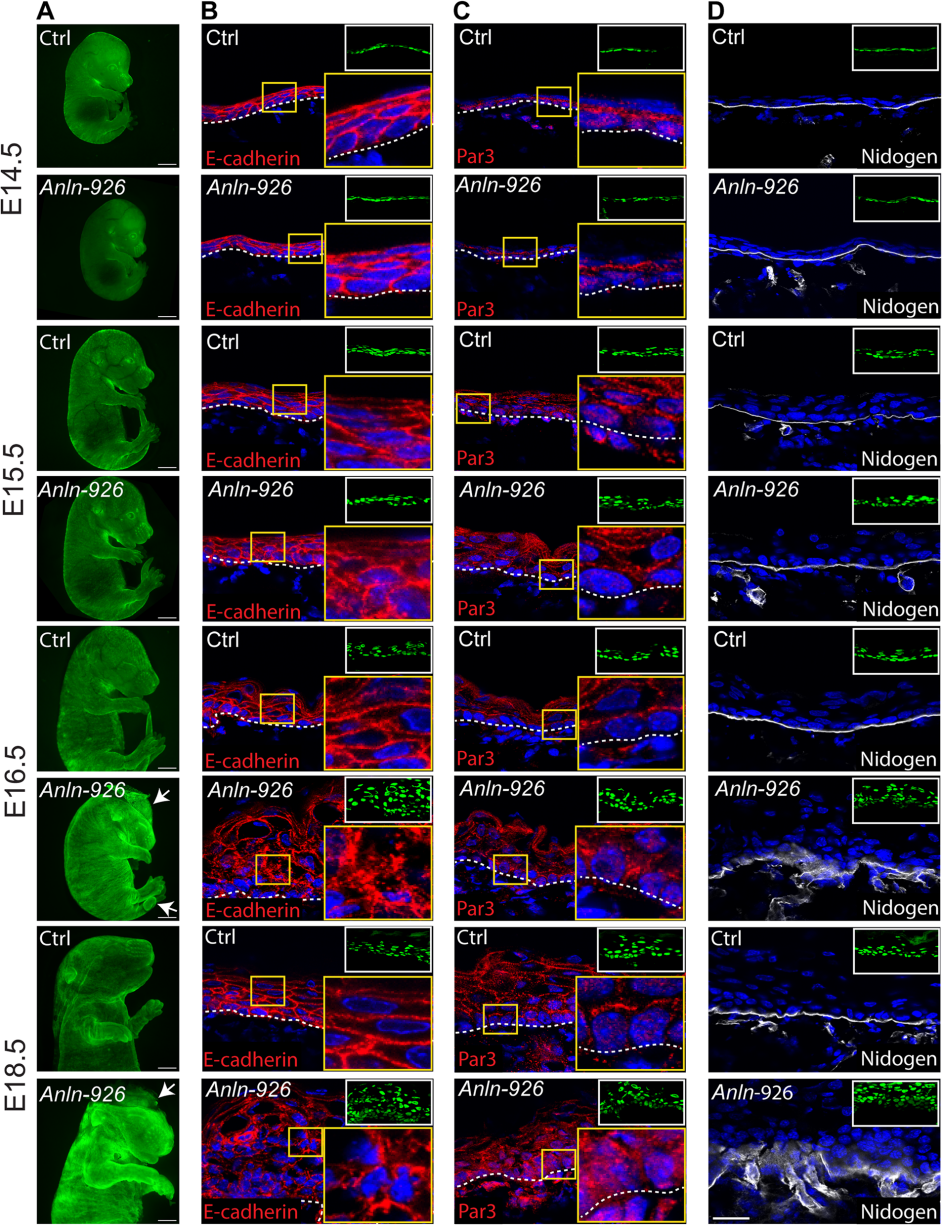
Adherens junction defects appear at E15.5 and escalate at E16.5 in *Anln*-depleted epidermis. **A** Stereomicroscopic images of E14.5, E15.5, E16.5, and E18.5 embryos infected on E9 with *shScr;H2B-GFP* (Ctrl) or *shAnln-926;H2B-GFP* lentiviruses. Arrows denote defects in the head and tail. Scale bar = 2 mm. **B** Sagittal views of 10-μm sections of dorsal skin from control and *shAnln-926* KD E14.5, E15.5, E16.5, and E18.5 embryos. Sections were immunostained for the adhesion receptor E-cadherin. **C** Dorsal skin sections from embryos treated as in **B** and immunostained for the polarity protein Par3. **D** Dorsal skin sections from embryos treated as in **B** and immunostained for the basement membrane protein nidogen. Dotted lines indicate the dermal–epidermal border. Insets show the transduced cells (H2B−GFP+). Yellow-framed insets show magnification of the boxed area. Nuclei were stained with DAPI (blue). Scale bars = 20 μm

Our evaluation of the surface anatomy of *shScr-* (control) and *shAnln*-transduced embryos showed that while E14.5 and E15.5 *Anln* knockdown (KD) embryos were comparable to control embryos, both E16.5 and E18.5 *Anln* KD embryos exhibited a curved tail, short limbs, and paucity of skin in the head region (Fig. 2A).

Because ANLN was recently shown to function in adherens junctions (AJs) [34, 36], and because we detected ANLN also in epidermal junctions (Fig. 1A, B), we asked whether ANLN deficiency affects AJ organization. Indeed, back skins labeled for E-cadherin (Fig. 2B), α-catenin, and β-catenin (Additional file 1: Fig. S1) showed a notable difference between control and *Anln-depleted* epidermis. While in the E14.5 epidermis of both control and *Anln*-depleted embryos, the three adhesion proteins were detected in the cell periphery, at E15.5, *Anln*-depleted epidermis featured small foci in which the distribution of the adhesion proteins was irregular (Fig. 2B and Additional file 1: Fig. S1). This trend worsened in E16.5 and E18.5 *Anln*-depleted epidermis, marked by the severely fragmented and discontinuous distribution of the three proteins (Fig. 2B and Additional file 1: Fig. S1).

Because cell adhesion is essential for apicobasal polarity [1, 49], we asked whether ANLN deficiency affects apicobasal polarity. To this end, back skins were labeled for the polarity protein Par3 (Fig. 2C). In E14.5 and E15.5 embryos, Par3 was enriched in the apical part of basal-layer cells in both control and *Anln* KD epidermis. Yet in E16.5, defects were detected in Par3 apical enrichment in *Anln*-depleted epidermis, and in E18.5 *Anln*-depleted epidermis, Par3 was diffuse (Fig. 2C).

Given the known link between BM organization, cell adhesion, and cell polarity [7], we assessed whether ANLN deficiency affects BM organization by staining control and *Anln*-depleted epidermis for the BM protein nidogen. In control and *Anln* KD skins from E14.5 and E15.5, nidogen formed a thin, continuous line between the dermis and epidermis. However, in E16.5 and E18.5 *Anln* KD epidermis, nidogen was diffused and discontinuous (Fig. 2D). Moreover, in the epidermis of E18.5 control embryos, β1 and β4 integrin were detected at the basal layer of the epidermis. β4 integrin was enriched at the basal part of the cells, and β1 integrin was detected throughout the cortex. However, in *Anln*-depleted epidermis, β4-integrin was diffused, and β1 integrin was detected in up to five layers (Additional file 2: Fig. S2).

All the defects mentioned above in cell adhesion, polarity, and BM organization seen in *Anln-926* embryos were recapitulated in embryos transduced with a second *Anln* shRNA (*Anln-2981*; Additional file 3: Fig. S3).

The above analyses demonstrated that the *Anln*-depleted epidermis is thicker than the control epidermis (Fig. 2, S1, and S2). Indeed, IF analysis in control E18.5 epidermis showed that the cell proliferation marker, Ki67, was restricted to the basal layer of the epidermis; however, in *Anln*-depleted embryos, Ki67 was detected in up to 5–6 layers (Additional file 4: Fig. S4). Nevertheless, *Anln* depletion did not hinder epidermal differentiation or the establishment of barrier function (Additional file 4: Fig. S4).

Together, these results demonstrate that the earliest defects in AJ organization emerge at E15.5; at E16.5 and E18.5, the adhesion defects escalate, and the tissue also exhibits defects in BM organization and apicobasal polarity.

### AJ organization is perturbed upon *Anln* KD in keratinocytes

**Fig. 3:**
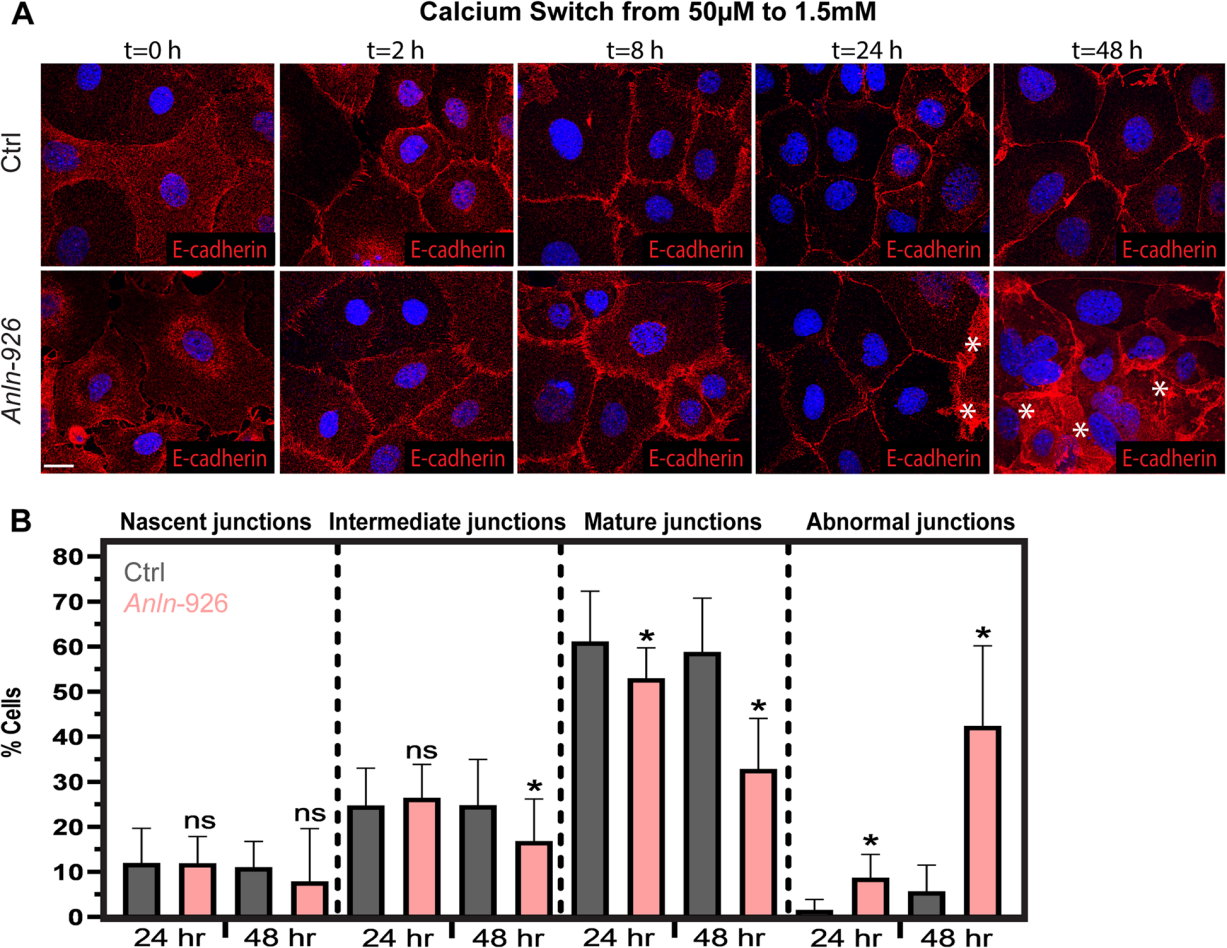
ANLN depletion hinders adherens junction assembly in cultured keratinocytes. **A** *shScr*- (Ctrl) and *shAnln*-926-transduced primary mouse keratinocytes (1^0^MKs) were induced to form adherens junctions by switching from low-calcium (50 μM) to high-calcium (1.5 mM) media and then immunolabeled for E-cadherin at the indicated time points. Asterisks denote abnormal junctions. Nuclei were stained with DAPI (blue). Scale bars = 20 μm. **B** Quantification of adherens junction organization from the data shown in **A**. Twenty-four hours after calcium switch, Ctrl, *n* = 35 fields; *shAnln-926*, *n* = 23 fields; from three independent experiments. For mature junctions, *P* = 0.001. For abnormal junctions, *P* < 0.0001. Forty-eight hours after calcium switch, Ctrl, *n* = 16 fields; *shAnln-926*, *n* = 17 fields; from three independent experiments. For intermediate junctions, *P* = 0.0259. For mature junctions, *P* < 0.0001. For abnormal junctions, *P* < 0.0001. All analyses were by unpaired two-tailed *t*-test

To probe further the defect in AJ organization in *Anln*-depleted epidermis, we turned to 1°MKs, which allow a more detailed analysis of AJ assembly and organization. Cultured 1°MKs were transduced with lentiviruses encoding *shScr* (control) or *shAnln*, and AJ assembly was induced by increasing the medium calcium concentration from 50 μM to 1.5 mM [50–52]. In both control and *Anln* KD 1°MKs, immunostaining of cells for E-cadherin showed that AJs were organized in filopodia-like protrusions, also known as nascent AJs, 2 h after calcium switch (Fig. 3A) and predominantly in a wide band at the cell periphery, also known as an intermediate junction, 8 h after calcium switch. Twenty-four hours after the calcium switch, in both control and *Anln* KD 1°MKs, the majority of AJs were organized into a narrow belt at the cell periphery, known as mature AJs; however, we detected a 13% decrease in mature junctions in *Anln*-depleted cells. Moreover, ~9% of the junctions in *Anln* KD 1°MKs E-cadherin had diffuse and structurally irregular shapes that we classified as “abnormal junction” (Fig. 3B). Interestingly, 48 h after the calcium switch, the majority, 42±17% of junctions in *Anln* KD 1°MKs were abnormal versus 6±6% in control cells. Together, these results demonstrate that while subtle defects exist in AJ assembly, during the first 24 h, AJ maintenance is severely disrupted in *Anln*-depleted 1°MKs.

### *Anln*-depleted epidermis exhibits heightened F-actin levels

**Fig. 4:**
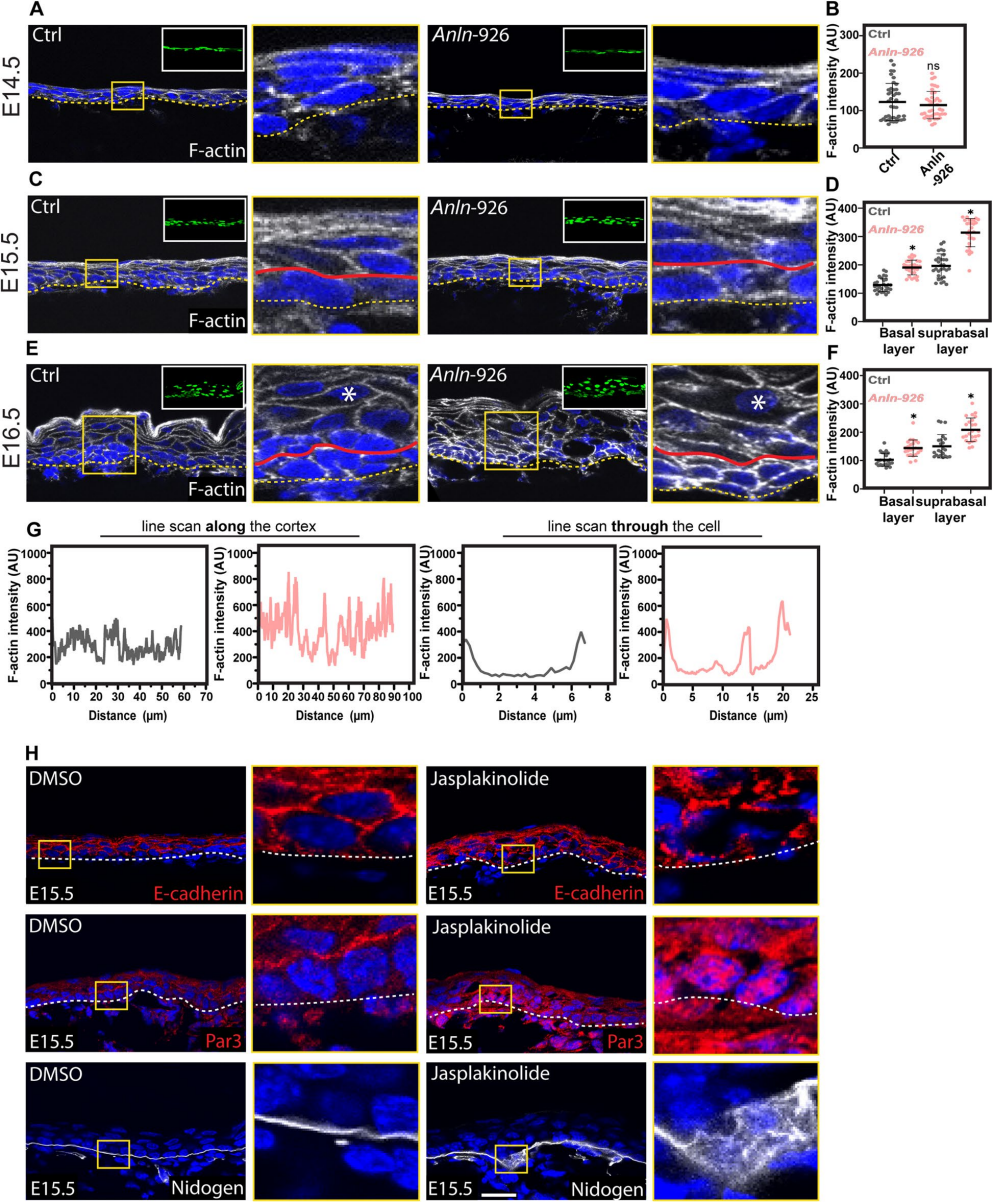
ANLN loss-of-function enhances F-actin levels in the developing epidermis. **A**, **C**, **E** Sagittal views of 10-μm sections of dorsal skin from *shScr* (ctrl) and *shAnln-926* transduced E14.5 (**A**), E15.5 (**C**), and E16.5 (**E**) embryos. Sections were stained for F-actin. **B** Quantification of F-actin staining intensity from the data shown in **A**. Horizontal bars represent the mean F-actin intensity, and circles represent individual microscopy fields. *n* = 4 embryos per condition. NS denotes not significant. **D** Quantification of F-actin staining intensity from the data shown in **C**. Horizontal bars represent the mean F-actin intensity, and circles represent individual microscopy fields. *n* = 3 embryos per condition. *P* = 0.0136 for Ctrl versus *Anln-926* basal layer; *P* = 0.0254 for Ctrl versus *Anln-926* suprabasal layers by unpaired two-tailed *t*-test. **F** Quantification of F-actin staining intensity from the data shown in **E**. Horizontal bars represent the mean F-actin intensity, and circles represent individual microscopy fields. *n* = 3 embryos per condition. *P* = 0.0123 for Ctrl versus *Anln-926* basal layer; *P* = 0.0392 for Ctrl versus *Anln-926* suprabasal layer by unpaired two-tailed *t*-test. **G** Line scan analyses from data shown in **E**. Asterisks denote analyzed cells. **H** Sagittal views of 10-μm sections of dorsal skin from wild-type E15.5 embryos treated with DMSO (Ctrl) or jasplakinolide, fixed, and immunolabeled for E-cadherin, Par3, and nidogen. Dotted lines in **A**, **C**, **E**, and **H** indicate the dermal–epidermal border. Insets in **A**, **C**, and **E** show the transduced cells (H2B−GFP+). Yellow insets in **A**, **C**, **E**, and **H** show magnification of boxed areas. Red lines in **C** and **E** indicate the boundary between basal and suprabasal layers. Nuclei were stained with DAPI (blue). Scale bars = 20 μm

Because cell-cell adhesion, polarity, and BM organization rely on the actin cytoskeleton, and because ANLN is an actin-binding protein [23, 24], we asked whether *Anln* depletion alters the actin cytoskeleton in the developing epidermis. To this end, we analyzed the levels of F-actin in the epidermis of E14.5, E15.5, and E16.5 control and *Anln*-depleted embryos. In the epidermis from E14.5 control embryos, F-actin was detected at the cell periphery in all the epidermal layers. Comparable levels and distributions of F-actin were detected in the epidermis of E14.5 *Anln*-depleted embryos (Fig. 4A, B). In E15.5 *Anln*-depleted embryos, F-actin’s distribution was normal compared to control. However, F-actin’s levels were upregulated in both basal and suprabasal layers (Fig. 4C, D). In E16.5 *Anln*-depleted embryos, F-actin levels remained abnormally high and its structural organization was disrupted. Line scan analysis demonstrated that F-actin levels along the cortex varied greatly, and high levels were also detected in the cytoplasm (Fig. 4E–G).

To determine whether the increase in F-actin levels can explain the defects in AJ organization, apicobasal polarity, and BM organization, we treated E15.5 embryos with DMSO (control), or jasplakinolide, a drug that increases the F-actin content [53]. Immunostaining for E-cadherin, Par3, and nidogen confirmed that increased F-actin levels are not compatible with normal AJ structure, apicobasal polarity, and BM organization (Fig. 4H).

### ANLN activity is essential for cytokinesis in cultured keratinocytes but not in the developing epidermis

**Fig. 5:**
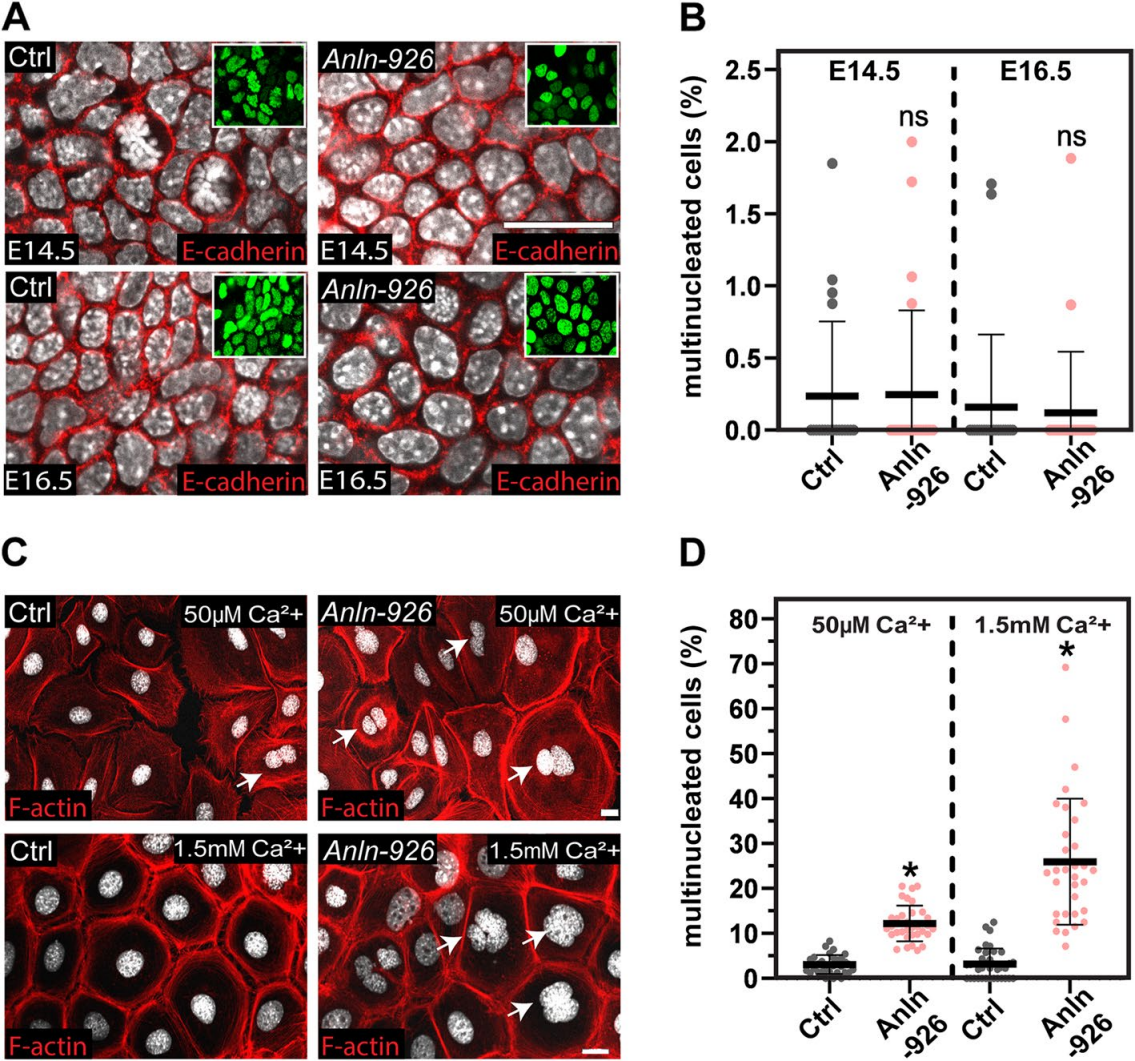
ANLN is essential for cytokinesis in cultured cells but not in the epidermis. **A** Whole-mount immunofluorescence images of control (Ctrl) and *shAnln-926*-transduced E14.5 and E16.5 embryos immunolabeled for E-cadherin and imaged at the middle of the basal layer. **B** Quantification of multinucleated cells from the data shown in **A**. *n* = 20 and 23 Ctrl and *shAnln-926*-transduced microscopic fields, respectively, from three E14.5 embryos per condition. *n* = 21 and 23 Ctrl and *shAnln-926* microscopic fields, respectively, from three E16.5 embryos per condition. Horizontal bars represent the mean, and circles represent individual cells. NS denotes not significant. **C** Wild-type keratinocytes were transduced with *shScr* (Ctrl) or *shAnln-926*, cultured in low- (50μM) and high-calcium media (1.5mM), and labeled for F-actin. **D** Quantification of multinucleated cells from the data shown in **C**. Low-calcium media: *n* = 31 and *n* = 34 ctrl and *Anln-926* microscopic fields from three independent experiments. *P* < 0.0001 by unpaired two-tailed *t*-test. High-calcium: *n* = 36 and *n* = 34 ctrl and *Anln-926* microscopic fields; from three independent experiments. *P* < 0.0001 by unpaired two-tailed *t*-test. Horizontal bars represent the mean, and circles represent the percentage of multinucleated cells in every microscopic field. Insets in **A** show the transduced cells (H2B−GFP+). Nuclei were stained with DAPI (grayscale). Scale bars = 20 μm

Because ANLN was shown to be essential for cytokinesis in many experimental systems [review in [27, 28, 32, 33]], we asked whether *Anln* depletion hinders cytokinesis in the developing epidermis. We analyzed two time points: E14.5, before defects in cell adhesion, polarity, and BM organization can be detected, and E16.5, when the processes mentioned above go awry and severe defects in embryo appearance can be seen (Fig. 2). To this end, we immunolabeled E14.5 and E16.5 control and *Anln*-depleted embryos for E-cadherin and DAPI and quantified the number of cells that had more than one nucleus in the basal layer of the epidermis [16]. In the epidermis from E14.5 and E16.5 control embryos, very few cells had more than one nucleus [54]. Comparable numbers of multinucleated basal cells were detected in the epidermis of *Anln*-depleted embryos (Fig. 5A, B).

Interestingly, in RhoA knockout mice, cytokinesis occurs in the epidermis but fails in cultured keratinocytes [55]. To determine whether ANLN plays a role in cytokinesis in vitro, 1°MKs were transduced with lentiviruses encoding *shScr* (control) or *shAnln*, cultured in low- or high-calcium media (50μM and 1.5mM respectively), and immunostained for F-actin and DAPI. In control 1°MKs, ~3% of the cells had more than one nucleus in both conditions. However, *Anln* loss-of-function increased the number of multinucleated cells. When cells were cultured in low-calcium media, we detected a 4-fold increase in the number of multinucleated cells (3±2.1 vs. 12±4) (Fig. 5C, D). When cells were cultured in high-calcium media, we detected an 8-fold increase in multinucleated cells (3.2±3.5 vs. 26±14) (Fig. 5C, D). Together, these results demonstrate that ANLN is essential for cytokinesis in vitro but not in vivo.

### ANLN activity is essential for mitotic rounding

Thus far, our results demonstrate that depletion of *Anln* progressively hinders epidermal architecture by affecting F-actin levels and organization, cell adhesion, polarity, and BM organization. To determine whether ANLN functions in other processes, we focused our attention on E14.5 embryos, in which the defects mentioned above are not detectable.

**Fig. 6:**
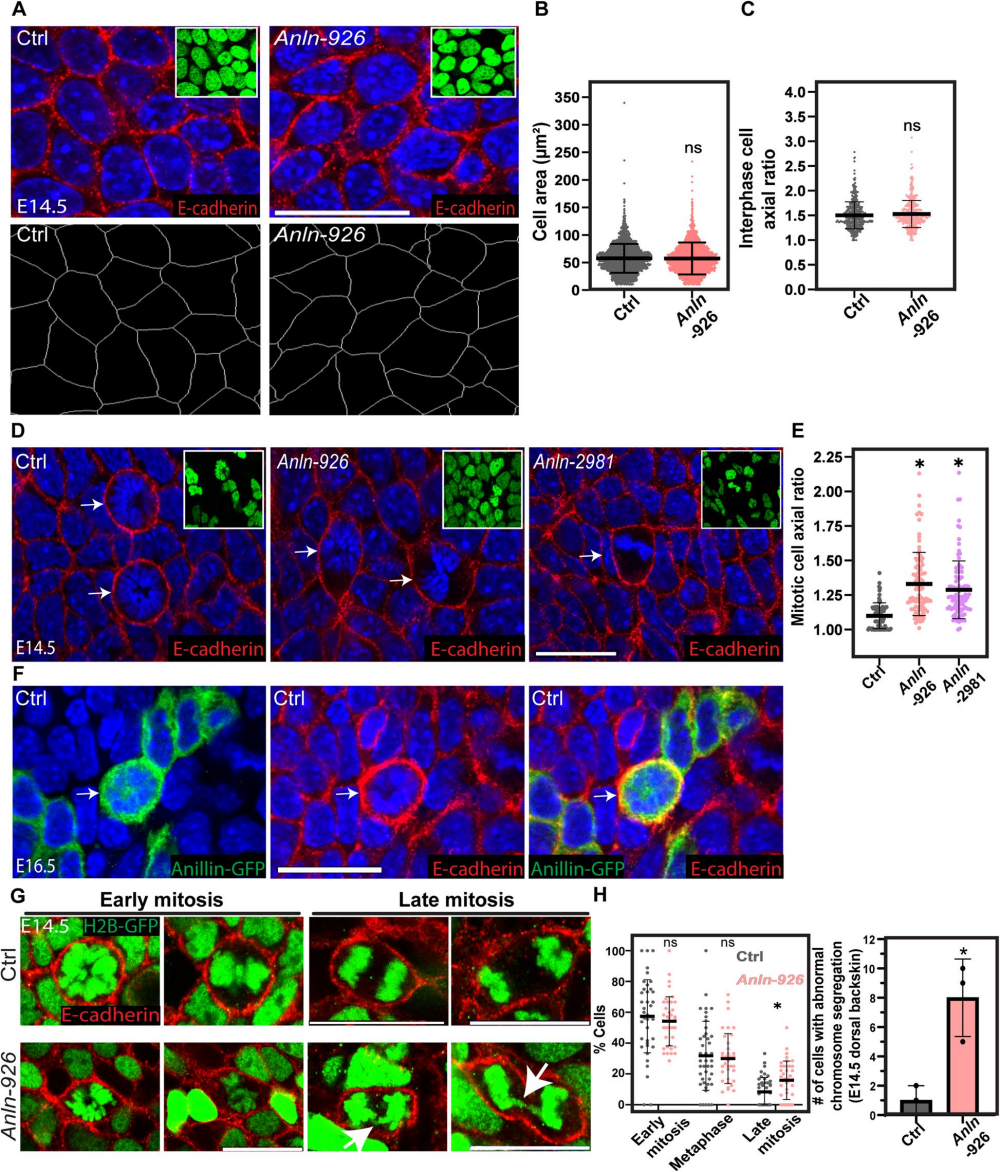
ANLN is essential for mitotic rounding early in the developing epidermis. **A** Upper panel: whole-mount immunofluorescence images of control (Ctrl) and *shAnln-926*-transduced E14.5 embryos immunolabeled for E-cadherin and imaged at the middle of the basal layer. Lower panel: Computer segmentation of E-cadherin staining to visualize cell borders. **B** Quantification of cell areas from the data shown in **A**. *n* = 2746 and 2759 Ctrl and *shAnln-926*-transduced cells, respectively, from three embryos per condition. Horizontal bars represent the mean, and circles represent individual cells. NS denotes not significant. **C** Quantification of axial ratio from the data shown in A. *n* = 539 and 560 Ctrl and *shAnln-926*-transduced cells, respectively, from three embryos per condition. Horizontal bars represent the mean, and circles represent individual cells. NS denotes not significant. **D** Whole-mount immunofluorescence of control and *Anln-926* KD E14.5 embryos immunostained for the adhesion receptor E-cadherin and imaged at the middle of the basal layer. Arrows denote early mitotic cells. **E** Quantification of the early mitotic cell axial ratio from the data shown in **D**. *n* = 77, 95, and 99 Ctrl, *Anln-926-* and *Anln-2981*-transduced cells, respectively, from four embryos per condition. Horizontal bars represent the mean, circles represent individual cells. *P* < 0.0001 for Ctrl versus *Anln-926*; *P* < 0.0001 for Ctrl versus *Anln-2981* by unpaired two-tailed *t*-test. **F** Whole-mount immunofluorescence images of *Anln-GFP*-transduced E16.5 embryos immunolabeled for E-cadherin and imaged at the middle of the basal layer. Arrows denote early mitotic cells. **G** Whole-mount samples were treated as in **A**. H2B-GFP (green) indicates infected cells. Arrows denote cells with abnormal chromosome segregation. **H** Quantification of the mitotic stage from the data shown in **G**. Horizontal bars represent the mean, and circles represent individual microscopic fields. *n* = 42 and 39 Ctrl and *Anln-926* microscopic fields, respectively, from four embryos per condition. *P* = 0.003 by unpaired two-tailed *t*-test. **I** Quantification abnormal chromosome segregation from the data shown in **G**. *n* = 3 embryos per condition*. P* = 0.0319 by unpaired two-tailed *t*-test. Horizontal bars represent the mean ± SD, and circles represent the mean of cells with abnormal chromosome segregation in 0.5cm^2^ dorsal skin E14.5 embryos. In **A** and **D**, insets show the transduced cells (H2B−GFP+). Nuclei were stained with DAPI (blue). Scale bars = 20 μm

In the epidermis and in cultured keratinocytes, cell-shape dynamics were shown to impact tissue architecture and cell fate [6, 10, 14, 16, 56–58]. Because ANLN is a key regulator of the actin cytoskeleton, which regulates cell shape [59] [60], we asked whether ANLN loss-of-function epidermis exhibits early defects in cell shape. To examine this, we used E-cadherin immunolabeling to delineate the epidermal cell borders of control and *Anln*-depleted E14.5 embryos. Our analysis of basal layer cell shape demonstrated that control and *Anln*-depleted epidermis had a comparable cell area (57.7 ± 26.3 vs. 57.4 ± 29 μm^2^) and axial ratio that represents cell elongation (1.5 ± 0.27 vs. 1.53 ± 0.27) (Fig. 6A–C).

Cell division is a process that involves extensive cell-shape changes. As cells enter mitosis, they round to facilitate division [review in [61–64]]. To determine whether ANLN activity affects mitotic rounding, we measured the axial ratio of early mitotic cells in the basal layer of E-cadherin-labeled E14.5 epidermis. The average axial ratio of control cells was ~1.1. However, in *shAnln-926-* and *shAnln-2981*-transduced embryos’ early mitotic cells, the axial ratio significantly increased to ~1.3 (Fig. 6D, E). Because mitotic rounding relies on the cortical cytoskeleton, we confirmed that ANLN-GFP was detected also at the cell periphery of early mitotic cells in the developing epidermis (Fig. 6F).

Together, our results demonstrate that during early epidermal development ANLN is dispensable for interphase cell shape; however, it is essential for mitotic rounding.

### ANLN depletion hinders mitotic-rounding-dependent processes

Mitotic rounding is essential for cell division, as its malfunction may hinder spindle morphology or orientation [65, 66] and prolong mitosis by hampering chromosome segregation [67–70]. We, therefore, asked whether the above-mentioned mitotic-rounding-dependent processes are altered in *Anln*-depleted epidermis.

Staining for acetylated tubulin and pericentrin to detect mitotic spindles revealed normal spindle morphology in the epidermis from E14.5 *Anln*-depleted embryos (Additional file 5: Fig. S5). To determine whether spindle orientation is affected by ANLN loss-of-function, we examined the expression of survivin, which labels the cleavage furrow in late-mitotic cells, and then calculated the angle between the two daughter nuclei and the BM [71, 72]. Spindle orientation analyses were done in E16.5 embryos, the earliest time point when spindle orientation becomes bimodal [73]. As expected, in the control epidermis, the vast majority of divisions were parallel (0 to 15°) or perpendicular (75 to 90°) to the BM, and only 7% of the divisions were oblique (15 to 75°) [71]. In contrast, *Anln* depletion caused an ~10-fold increase in the number of oblique divisions (Additional file 4: Fig. S4), suggesting that spindle orientation goes awry without ANLN activity. However, it is noteworthy that mitotic rounding, cell adhesion, and apicobasal polarity are well known to play a role in spindle orientation, and all these processes are abnormal in E16.5 *Anln*-depleted epidermis, making it challenging to interpret proximate causes for the orientation defects.

To determine whether ANLN loss-of-function alters the progression through mitosis, E14.5 control and *Anln* KD embryos were labeled for E-cadherin and imaged at the basal layer, and the number of early mitotic (prophase and prometaphase), metaphase, and late mitotic (anaphase and telophase) cells was determined for every microscopic field. In control and *Anln* KD epidermis, we detected a comparable number of cells in early mitosis and metaphase. However, we noted an 90% increase in the number of cells in late mitosis (Fig. 6G–H). Moreover, careful inspection of late-mitosis cells revealed abnormal chromosome segregation in *Anln-*depleted embryos (Fig. 6G). Quantification of the number of late mitotic cells in which abnormal chromosome segregation was detected in the epidermis of E14.5 embryos revealed an 8-fold increase in their prevalence in *Anln*-depleted epidermis (1±1 vs. 8±2.6) (Fig. 6I).

### ANLN regulates cortical actomyosin levels and distribution in early mitotic cells

**Fig. 7:**
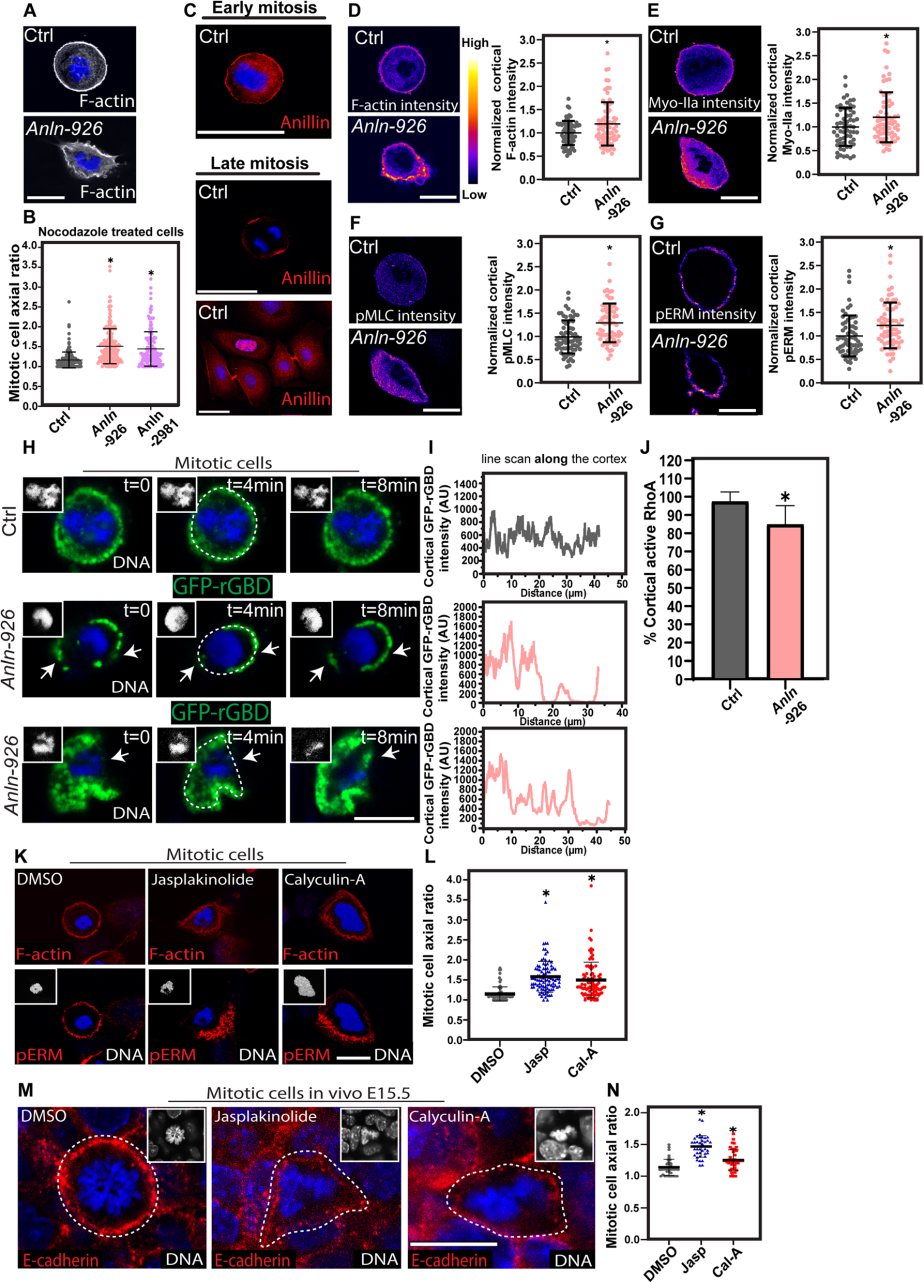
ANLN is essential for mitotic rounding in cultured keratinocytes. **A** Wild-type keratinocytes were transduced with *shScr* (Ctrl) or *shAnln-926*, treated with nocodazole for 6 hours, fixed, and labeled for F-actin. **B** Quantification of the early mitotic cell axial ratio from the data shown in **A**. Horizontal bars represent the mean, and circles represent individual cells. *n* = 181, 176, and 155 Ctrl, *Anln-926-*, and *Anln-2981*-transduced cells, respectively, from four experiments per condition. *P* < 0.0001 for Ctrl versus *Anln-926*, *P* < 0.0001 for Ctrl versus *Anln-2981* by unpaired two-tailed *t*-test. **C** Wild-type keratinocytes immunolabeled for ANLN. **D** Wild-type keratinocytes treated as in **A** and immunostained for F-actin. Quantification of the normalized cortical intensity is presented to the right of the image. *n* = 76 and 76 Ctrl and *Anln-*transduced cells, respectively, from three experiments. *P* = 0.0019 for Ctrl versus *Anln-926* by unpaired two-tailed *t*-test. **E** Wild-type keratinocytes treated as in **A** and immunostained for myosin IIa. Quantification of the normalized cortical intensity is presented to the right of the image. *n* = 62 and 67 Ctrl and *Anln-*transduced cells, respectively, from three experiments. *P* = 0.0148 by unpaired two-tailed *t*-test. **F** Wild-type keratinocytes treated as in **A** and immunostained for pMLC. Quantification of the normalized cortical intensity is presented to the right of the image. *n* = 70 and 68 Ctrl and *Anln-*transduced cells, respectively, from three experiments. *P* < 0.0001 for Ctrl versus *Anln-926* by unpaired two-tailed *t*-test. **G** Wild-type keratinocytes treated as in **A** and immunostained for pERM. Quantification of the normalized cortical intensity is presented to the right of the image. *n* = 64 and 68 Ctrl and *Anln-*transduced cells, respectively, from three experiments. *P* = 0.0061 for Ctrl versus *Anln-926* by unpaired two-tailed *t*-test. **D**–**G** Horizontal bars represent the mean, and circles represent individual cells. **H** Representative images of GFP-rGBD in *shScr* (ctrl) or *Anln-926*-transduced primary mouse keratinocytes. Arrows indicate the changes of active RhoA localization. Nuclei were labeled with Hoechst. The dotted line denotes cell edge. Scale bars = 10 μm. **I** Line scan analyses from data shown in **H**, *t* = 4 min. **J** Quantification of cortical coverage of GFP-rGBD in early mitotic cells. Horizontal bars represent the mean. *n* = 13 and 14 Ctrl and *Anln-*KD 1^0^MK, respectively, from three independent experiments. *P* = 0.0005 by unpaired two-tailed *t*-test. **K** Wild-type keratinocytes were co-treated with nocodazole and DMSO (Ctrl) or nocodazole and jasplakinolide, or nocodazole and calyculin A, fixed, and labeled for F-actin and pERM. **L** Quantification of mitotic cell axial ratio from the data shown in **K**. Horizontal bars represent the mean, and circles represent individual cell. *n* = 86, 93, and 93 DMSO, jasplakinolide, and calyculin A-treated cells, respectively, from three experiments. P<0.0001 for DMSO versus jasplakinolide; *P* < 0.0001 for DMSO versus Calyculin A by unpaired two-tailed *t*-test. **M** Whole-mount immunofluorescence images of wild-type E15.5 embryos treated with DMSO (Ctrl), jasplakinolide, or Calyculin A, fixed, and immunolabeled for E-cadherin and imaged at the middle of the basal layer. The dotted line denotes cell edge. **N** Quantification of early mitotic cell axial ratio from data shown in **M**. *n* = 39, 37, and 43 DMSO, jasplakinolide, and Calyculin A, respectively, from three treated embryos, per condition. Horizontal bars represent the mean, and circles represent individual cells. *P* < 0.0001 for DMSO versus jasplakinolide; *P* = 0.0034 for DMSO versus Calyculin A by unpaired two-tailed *t*-test. In **H**, **K**, and **M**, insets show the nuclei in a grayscale. Nuclei were stained with DAPI (blue). Scale bars = 20 μm

Our results demonstrate that ANLN is essential for mitotic rounding and its dependent processes early in epidermal development. To probe deeper into the role of ANLN in mitotic rounding, we first asked whether the mitotic rounding defects can be recapitulated in cultured keratinocytes. To this end, control and *Anln*-depleted 1^0^MKs were treated with nocodazole to arrest early mitotic cells for axial ratio analyses [17]. The average axial ratio of *shScr*-transduced (control) mitotic 1^0^MKs was 1.16 ± 0.2%, while in *Anln-*depleted 1^0^MKs, the axial ratio markedly increased to ~1.5 (Fig. 7A, B). Defects in mitotic rounding were also detected when cells were treated with monastrol, which arrests mitosis by a distinct mechanism [74] (Additional file 6: Fig. S6). Next, we investigated whether ANLN can be detected in the cell cortex of early mitotic cultured cells. Immunolabeling of ANLN revealed high levels of the protein throughout the cortex of early mitotic cells (Fig. 7C). In late mitotic cells, ANLN was no longer detected throughout the cortex; its highest levels were detected in the cleavage furrow contractile zone and the midbody (Fig. 7C). Together, these data demonstrate that ANLN localizes at the cell cortex of early mitotic cells and that it is essential for mitotic rounding in vitro and in vivo.

Mitotic rounding relies on the extensive remodeling of the actomyosin cortical cytoskeleton. Two main pathways were shown to execute this process, the ECT2-RhoA pathway, which remodels F-actin and Myosin II, and an independent pathway in which slik kinase phosphorylates and activates the ERM proteins (ezrin, radixin, and moesin), which link F-actin to the membrane [65, 66, 75] [76]. Because the actin cytoskeleton regulates gene expression [77], we asked whether ANLN activity affects the overall levels of the key mitotic rounding proteins. To this end, we conducted western blot analyses in control and *Anln*-depleted 1^0^MKs that contain both mitotic and non-mitotic cells. We detected comparable levels of β- and γ-actin and myosin IIa. Yet, we observed an increase in phosphorylated myosin regulatory light chain (pMLC), which indicates active myosin II, and phosphorylated ERM proteins (pERM) in *Anln*-depleted cells (Additional file 7: Fig. S7).

Next, we treated control and *Anln*-depleted 1^0^MKs with nocodazole and analyzed the fluorescence intensity and distribution of the following cortical proteins specifically in the mitotic cortex: F-actin, myosin IIa, pMLC, and pERM. We detected a significant increase in the cortical intensity of all four proteins in *Anln* KD mitotic 1^0^MKs. Cortical F-actin levels increased by 19%, cortical myosin IIa levels by 20%, cortical pMLC levels by 33%, and cortical pERM levels by 23% (Fig. 7D–G). Moreover, while all the proteins in control cells were evenly distributed throughout the cell cortex, which appeared as a thin ring in the cell periphery, in *Anln*-depleted cells, the cortex was thicker than normal and the distribution of the cortical proteins was uneven, with distinct foci of high and low levels (Fig. 7D–G). A cumulative frequency distribution analysis confirmed a significant defect in the distribution of all the proteins mentioned above along the mitotic cortex (Additional file 7: Fig. S7).

Together, our results demonstrate that ANLN is a prominent component of the cortex of early mitotic cells; ANLN activity is essential for the control of the levels, distribution, and activity of the cortical actomyosin cytoskeleton.

### ANLN regulates active RhoA localization

Next, we explored how ANLN activity regulates the actomyosin cytoskeleton. Because RhoA orchestrates actomyosin remodeling during mitotic rounding [65, 66, 75], and because ANLN was shown to regulate RhoA function [31, 35], we assessed whether RhoA overall levels and activity are altered in *Anln*-depleted 1^0^MKs that contain both mitotic and non-mitotic cells. Western blot analyses and pull-down assays to detect total and active RhoA (RhoA-GTP) found comparable levels and activity of RhoA in control and *Anln*-depleted 1^0^MKs (Additional file 7: Fig. S7).

Next, we asked whether ANLN affects active RhoA localization. To this end, we transfected control and *Anln*-depleted 1^0^MKs with GFP-rGBD, an active RhoA sensor [78]; treated the cells with nocodazole; and analyzed their distribution with time-lapse microscopy in mitotic cells. In control mitotic cells, GFP-rGBD covered 98±5% of the cortex and its levels along the cortex fluctuated by 3–4-folds (Fig. 7H–J). In sharp contrast, in *Anln*-depleted cells, GFP-rGBD covered 84±10% of the cortex and its levels in loci were it was detectable varied with to up to ~20–30-folds (Fig. 7H–J). Moreover, in many *Anln*-depleted cells, GFP-rGBD was detected over an abnormally broad region at the cell periphery (Fig. 7H).

Together, our results demonstrate that, while ANLN depletion does not hinder overall RhoA levels or activity, it severely alters active RhoA organization in the cortex of early mitotic cells.

### An increase in F-actin content or myosin II activity is not compatible with mitotic rounding and pERM localization

Defects in RhoA can explain abnormalities in the cortical actin and myosin levels, activity, and distribution. However, ERM activation during mitotic rounding is executed by a distinct pathway that does not involve RhoA or Rho-kinase [65]. To determine whether defects in mitotic rounding can be explained by an increase in F-actin content or myosin II motor activity, nocodazole-treated 1^0^MKs were co-treated with DMSO (control) or with jasplakinolide or calyculin A, drugs that increase the F-actin content [53] and enhance myosin II motor activity [79], respectively. Cells were then fixed and immunolabeled for F-actin and pERM. Calculations of the mitotic cell axial ratio showed that while the DMSO treatment did not hinder mitotic rounding (1.15±0.2), jasplakinolide and calyculin A treatments significantly increased the axial ratio by 37% and 30%, respectively (Fig. 7K, L). Moreover, both the jasplakinolide and calyculin A treatments disrupted F-actin and pERM organization. Similar to its distribution in *Anln* KD cells, cortical F-actin was thicker than normal and discontinuous, and pERM was absent from much of the cortex (Fig. 7L). Additionally, DMSO (control), jasplakinolide, and calyculin A treatments in E15.5 embryos confirmed that an increase in F-actin content or myosin II activity is incompatible with mitotic rounding in vivo (Fig. 7M, N).

Collectively, the results presented here demonstrate that ANLN is a major regulator of epidermal morphogenesis. Early in epidermal development, the defects are restricted to cells that undergo mitotic rounding, which requires extensive cytoskeletal remodeling. Later in development, cytoskeletal defects also impact interphase cells and hinder cell adhesion, polarity, and BM organization.

## Discussion

**Fig. 8:**
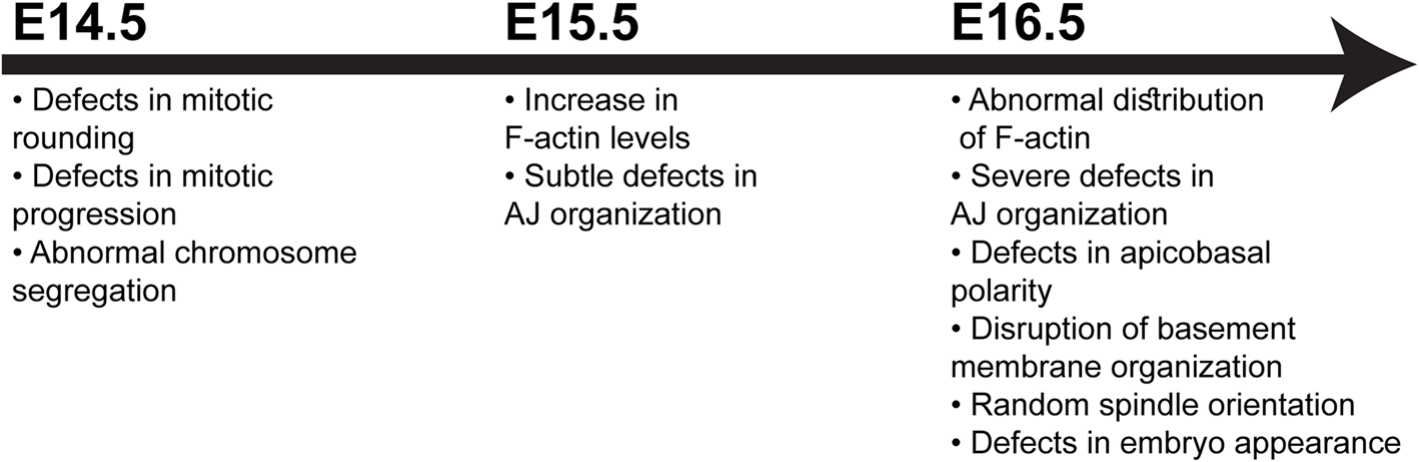
ANLN is essential for epidermal morphogenesis. A summary of the temporal progression of the *Anln* KD phenotype

Tissue development requires tight spatiotemporal coordination between processes that control cell shape, proliferation, and differentiation [1, 3, 9]. However, the mechanisms that orchestrate these processes are poorly understood. Our study identifies ANLN as a major regulator of the actin cytoskeleton and epidermal morphogenesis. In keeping with previous reports [31, 34–36], we demonstrate that ANLN is essential for AJ organization in the developing epidermis and defects in the actin cytoskeleton can also explain abnormalities in apicobasal polarity and BM assembly [15], which are all major regulators of epidermal morphogenesis [3, 80]. Moreover, we uncover an early, novel function for ANLN in mitotic rounding and its dependent processes: chromosome segregation, spindle orientation, and mitotic progression (Fig. 8). The early timepoint in which mitotic rounding goes awry in the absence of ANLN function, before any other defect, reflects the pivotal role of ANLN in mitotic rounding.

Our results demonstrate that ANLN is a major regulator of cell-cell adhesion in the developing epidermis. Comparable adhesion defects were reported in additional experimental systems in which ANLN’s function was compromised, including Xenopus embryos [35, 36], podocytes [39, 81], and human prostate, colonic, and lung cells [82]. However, despite the functional similarity, it is noteworthy that the actin structures supporting cell-cell adhesions were differentially affected by the ANLN loss-of-function. In the Xenopus embryos and in cultured human cells, which are all simple epithelia, ANLN depletion resulted in a decrease in actomyosin levels and contractile activity of the apical actomyosin belt [36, 82] and the medial-apical actomyosin network [35]. In the epidermis, a stratified epithelium, polarity is manifested at the tissue level across many layers rather than at the level of single cell [1, 2, 11]. We found that the absence of ANLN activity caused cortical actomyosin levels to be upregulated and the epidermis’s structural organization to be severely disrupted. These dissimilarities may result from the differences mentioned above at the molecular, cellular, and tissue architecture levels. Alternatively, the differences may reflect the complex biology of ANLN, which can be detected in different cellular compartments [23, 26] and can interact with regulators that execute opposing cellular activities [26, 28]. These alternative possible explanations emphasize the need to explore the function of ANLN in a variety of experimental systems to fully understand its complex biology.

Interestingly, our results demonstrate that ANLN activity is essential for cytokinesis in cultured keratinocytes but not in the developing epidermis. Similar results were obtained when ANLN binding partner, RhoA, was deleted in vitro and in vivo [55]. While numerous factors differentiate between physiological and non-physiological conditions, a striking difference exists in the mechanical environment [83] and in cell shape [6, 14, 16], both of which are known to impact on actin-based processes and the activity of motor proteins [84–86]. Moreover, our results also show that the conditions in which *Anln* KD 1^0^MK are cultured impact the execution of cytokinesis. While cytokinesis failed when cells were cultured in low- and high-calcium conditions, the number of multinucleated cells significantly increased when calcium levels were high. However, also in this experimental system, calcium signals affect many cellular processes, including keratinocyte adhesion and differentiation, processes that involve profound changes in cell shape and the cytoskeleton [6]. Additional work is required to understand better how the cellular environment alters the requirement for ANLN activity during cytokinesis.

In the current study, we show, for the first time, to our knowledge, that ANLN is essential for mitotic rounding in vitro and in vivo. The notion that ANLN is a key regulator of mitotic rounding, and its related processes, has profound consequences for tissue development and health. Mitotic rounding contributes to diverse morphogenetic processes such as cell rearrangements during gastrulation [87], lumen formation [88], epithelial sheet invagination [89], and intestinal villi formation [90]. Mitotic rounding functions in morphogenesis by exerting forces that alter tissue shape and inducing remodeling of cell adhesion in neighboring cells [91] and by regulating spindle orientation that localizes daughter cells in the developing tissue [92]. Spindle orientation also functions in cell-fate specification, and its malfunction hinders epidermal differentiation [71, 93].

The role of mitotic rounding in cancer is particularly interesting. Intriguingly, increased ANLN levels correlate with poor prognosis in many types of cancer, including breast, non-small cell lung, pancreatic [94], colorectal [42], and many others [reviewed in [25]]. To undergo mitotic rounding in the stiff tumor environment [95–97], cancer cells upregulate the levels of key components of mitotic rounding [96, 97]. It is tempting to speculate that ANLN contributes to the malignant phenotype by enhancing mitotic rounding and allowing cancer cells to divide.

## Conclusions

Overall, the current study demonstrates that ANLN plays a crucial role in skin biology. Our results highlight a novel role for ANLN in mitotic rounding and demonstrate how the cortical cytoskeleton orchestrates cell division, adhesion, polarity, BM organization, and morphogenesis in a mammalian organ in vivo.

## Materials and methods

### Mice and primary mouse keratinocytes

All experimental protocols were approved by the Tel Aviv University Animal Care and Use Committee. Hsd:ICR (CD1) mice (Envigo) were used for all experiments. Epidermal keratinocytes were isolated as previously described [98]. Briefly, dorsal skin was removed from newborn mice and incubated with dispase (Sigma-Aldrich), followed by isolation of the epidermis which was treated with trypsin (Biological Industries). Keratinocytes were plated on fibroblast feeder cells for four passages and then plated in tissue culture dishes without feeder cells.

### Lentiviruses

Lentiviruses were produced as previously described [14, 46]. Briefly, lentiviral plasmids were generated by cloning oligonucleotides into pLKO.1-TRC (gift from David Root, Broad Institute, Cambridge, MA, USA; Addgene plasmid #10878) or LV-GFP (gift from Elaine Fuchs, Rockefeller University, New York, NY, USA; Addgene plasmid #25999) by digestion with EcoRI and AgeI, as described in the Genetic Perturbation Platform (GPP) website (http://portals.broadinstitute.org/gpp/public/resources/protocols).

shRNA sequences were obtained from GPP (http://portals.broadinstitute.org/gpp/public/):

Anln(926) construct #TRCN0000090265, target sequence 5′-CCGCTTGTTTATCCAAATCTT-3′

Anln(2981) construct #TRCN0000090264,target sequence 5′-GCAGCCTTCATTCTTCAGTTA-3′

pEGFP-Anillin was a gift from Micheal Glotzer (Addgene plasmid #68027, PMID 18158243).

### In utero lentivirus injection

Lentiviruses were injected into gestating mice as previously described [46].

Briefly, females at E9 were anesthetized with isoflurane and each embryo (up to six per litter) was injected with 0.4–1 μl of ∼2 × 10^9^ colony-forming units (CFU) of the appropriate lentiviruses. Controls were both uninfected littermates of *shAnln*-*926*/*2981*;H2B-GFP lentivirus-injected embryos and *shScr*;H2B-GFP lentivirus-injected embryos.

### In vitro lentivirus infection of keratinocytes

Primary mouse keratinocytes (1^0^MKs) were generated as described above and infected as previously described [46]. Briefly, 1°MKs were plated at 10^5^ cells/well in 6-well plates and infected with 250 μl of ∼10^7^ CFU lentiviruses (*shScr* or *shAnln*-*926/2981* with a puromycin resistance gene) in the presence of 100 μg/ml Polybrene (Sigma-Aldrich) for 48 h. Cells were then treated with 3 μg/ml puromycin (Sigma-Aldrich) for 72 h to select for infected cells. Selected cells were cultured with 1.5 μg/ml puromycin for an additional 24 h and then used in experiments.

### Semiquantitative RT-PCR

RNA was extracted from cells using a Direct-zol RNA extraction kit (Zymo Research; R2060), and equal amounts of RNA were reverse-transcribed using ProtoScript First Strand cDNA Synthesis Kit (New England Biolabs). Semiquantitative PCR was conducted using a StepOnePlus System (Thermo Fisher Scientific). Amplifications (40 cycles) were performed using the primers indicated below and cDNA template mixed with LightCycler DNA Master SYBR Green mix. The specificity of the reactions was determined by subsequent melting curve analysis. StepOnePlus software was used to adjust for background fluorescence. mRNA levels were quantified using the number of cycles needed to reach the crossing point according to the 2-delta CT method. Data are presented as mRNA levels of the gene of interest normalized to peptidylprolyl isomerase B (*Ppib*) mRNA levels. The primers were *Anln* forward 5′-acaatccaaggacaaacttgc-3′ and reverse 5′-gcgttccaggaaaggctta-3′; Ppib forward 5′-GTGAGCGCTTCCCAGATGAGA-3′ .

### Antibodies for western blot analysis and immunofluorescence

Antibodies against the following proteins were purchased and used as follows: GFP (Abcam, ab13970, 1:3000), keratin 14 (K14) (BioLegend, PRB-155P, 1:1000), keratin 10 (K10) (BioLegend,PRB-159P, 1:1000), loricrin (BioLegend, Poly19051, 1:1000), nidogen (Santa Cruz Biotechnology, sc-33706,1:2000), glyceraldehyde 3-phosphate dehydrogenase (GAPDH; Cell Signaling Technology, 5174, 1:1000), survivin (Cell Signaling Technology, 2808, 1:500), Par3 (Millipore, 07-330, 1:500), E-cadherin (Cell Signaling Technology, 3195, 1:500), and α-catenin (Sigma-Aldrich, C8114, 1:500), stable β-catenin (Cell Signaling Technology,19807, 1:800), pericentrin (BioLegend, PRB-432C, 1:500), Myosin-IIA (BioLegend, PRB-440P, 1:500), β-actin (Sigma-Aldrich, clone Ac15, 1:5000), γ-actin (Millipore, clone 2A3, 1:2000), phospho-MLC2 (Ser10) (Cell Signaling Technology, 3671,1:1000), phospho-ERM (Cell Signaling Technology, 1:200), Ac-tubulin (Sigma-Aldrich, 1:300), β4 integrin (BD Biosciences, clone 346-11A, 1:400), β1 integrin (Millipore, clone 12G10, 1:100), and Ki67 (Abcam, ab15580, 1:500). Anillin antibody was a kind gift from Michael Glotzer (University of Chicago, PMID: 18158243).

Secondary antibodies were of the appropriate species/isotype reactivity conjugated to Alexa Fluor 488, Alexa Fluor 647, or Rhodamine Red-X (Jackson Immuno-Research). F-actin was labeled with Phalloidin-iFluor 647 (Abcam, ab176759). Nuclei were labeled with 4′,6-diamidino-2-phenylindole (DAPI; Sigma-Aldrich).

### Immunofluorescence and western blotting

For immunofluorescence microscopy, embryos were embedded in OCT (Scigen), frozen, sectioned at 10 μM using a Leica CM1860 cryostat, and fixed in 4% formaldehyde for 10 min or in ice-cold methanol for 5 min. Sections were then blocked with 0.1% Triton X-100, 1% bovine serum albumin, 5% normal donkey serum in phosphate-buffered saline, or in MOM Basic kit reagent (Vector Laboratories). Sections were incubated with primary antibodies (see above) overnight at 4°C and with secondary antibodies for 1 h at room temperature. For whole-mount immunofluorescence microscopy, embryos were fixed for 1–3 h in 4% formaldehyde, and the dorsal skin was removed mechanically and stained as described above.

For western blot analysis, cells were lysed with RIPA buffer (Sigma-Aldrich) and proteins were quantified using a BCA kit (Pierce). Samples of 5–20 μg protein were separated by 12% SDS PAGE and transferred to nitrocellulose membranes. Membranes were blocked and incubated overnight at 4°C with primary antibodies to Anln (1:2000), GAPDH (1:1000), and then with horseradish peroxidase-conjugated antibodies (1:10,000 dilution in blocking solution) at room temperature for 1 h. Blots were developed using an Enhanced Chemiluminescence Detection Kit (Biological Industries) according to the manufacturer’s instructions. Images were obtained using a FUSION FX7 spectra imaging system.

### RhoA pull-down activity assay

Keratinocytes were infected with *shScr;puromycin* or *shAnln;puromycin*, selected with 3 μg/ml puromycin (sigma). Cells were then grown in 10-cm-in-diameter dishes for 2 days to 100% confluence, washed with ice-cold PBS, lysed, and processed for pulldown assays according to the manufacturer’s instructions (cytoskeleton, BK036).

### Confocal microscopy

Images were acquired with a Nikon C2+ laser-scanning confocal microscope using a 60×/1.4 oil objective or a 20×/0.75 air objective (Nikon). Images were recorded as 1024 × 1024 square pixels. RGB images were assembled in ImageJ software (imagej.nih.gov), and panels were labeled in Adobe Illustrator CC.

### Calcium switch experiments

Control and anillin-depleted 10MK were seeded either in high confluency (60,000 cells/well in 24-well plate, to study sheet assembly) in 24-well plate in low calcium (50 μM Ca2+) media. Upon reaching a confluent monolayer, the media were replaced with high-calcium media (1.5mM Ca2+). Twenty-four or 48 h after the calcium switch, cells were fixed and stained for E-Cadherin (Cell Signaling Technology, 3195, 1:500). Depending on the E-cadherin organization, the AJs were classified as nascent (E-cadherin in the form of filopodia like protrusions), intermediate (E-cadherin as a broad belt), mature junctions (E-cadherin as a narrow band at the junctions), or abnormal junctions (E-cadherin had diffuse and structurally irregular shapes).

### Quantification of cortical protein levels and axial ratio in cultured keratinocytes

Keratinocytes were infected with *shScr;puromycin* or *shAnln;puromycin*, selected with 3 μg/ml puromycin (sigma), and plated on fibronectin-coated coverslips (40,000 cells in a single well of a 24-well plate). Twenty-four hours later, at ~60% confluency, cells were treated with high-calcium (1.5mM Ca^2+^) media, supplemented with 8μM nocodazole (Sigma-Aldrich) for 6 h, and then fixed and labeled with Phalloidin-iFluor 647 (Abcam, ab176759), phospho-ERM (Cell Signaling Technology, 1:200), phospho-MLC (Ser10) (Cell Signaling Technology, 3671,1:1000), or Myosin-IIA (BioLegend, PRB-440P, 1:500) overnight at 4°C. After washing, sections were incubated with the appropriate secondary antibody (1:500 dilution) at room temperature for 1 h. Data was collected using a Nikon C2+ 60×/1.4 objective that generates optical sections of 0.49 μm at the middle of the cell. The cortical intensity was measured with the “freehand line tool” (ImageJ) with a width of 5 pixels. The mean gray value of *Anln* KD cells was normalized to that of the control cells. The same raw data was used to measure the axial ratio, using the “fit ellipse” tool (ImageJ).

### Quantification of cell shape in vivo

Embryos were injected with lentiviruses encoding *shScr; H2B-GFP* or *shAnln;* and *H2B-GFP* on E9 and harvested at E14.5. Whole-mount samples were labeled for E-cadherin overnight at 4°C. After washing, sections were incubated with a secondary antibody at room temperature for 2 h. Data were collected using a Nikon C2+ 60×/1.4 objective that generates optical sections of 0.49 μm at the middle of the basal layer. Images were filtered using a two-dimensional band-pass filter and segmented based on E-cadherin staining using the tissue analyzer plug-in in ImageJ. Packing analyzer software v2 [99] software was used to measure cell area. From the same raw data, the axial ratio was calculated using the “fit ellipse” tool in ImageJ.

### Quantification of spindle orientation

Spindle orientation was quantified as previously described [71]. Briefly, embryos were injected with lentiviruses encoding *shScr;H2B-GFP* or *shAnln;H2B-GFP* on E9 and harvested at E16.5. Embryos were frozen in OCT, sectioned (10 μm), fixed, and incubated with an anti-survivin antibody (1:500) overnight, followed by incubation with a secondary antibody at room temperature for 1 h. Images were collected using a Nikon C2+/60X/1.4 objective and the angle between the two daughter nuclei and the BM was calculated using the “angle” tool in ImageJ.

### Quantification of multinucleated cells in vivo and in vitro

Embryos were injected with lentiviruses encoding *shScr;H2B-GFP* or *shAnln;H2B-GFP* on E9 and harvested at E14.5. Whole-mount samples were immunolabeled for E-cadherin antibody (1:500) overnight at 4°C, followed by incubation with a secondary antibody and DAPI at room temperature for 2 h. Data were collected using a Nikon C2+ 60×/1.4 objective that generates optical sections of 0.49 μm at the middle of the basal layer.

In vitro, keratinocytes were infected with *shScr;puromycin* or *shAnln;puromycin*, selected with 3 μg/ml puromycin (Sigma), and plated on fibronectin-coated coverslips (60,000 cells in a single well of a 24-well plate). When cells created a confluent monolayer, the media were replaced with high-calcium media (1.5mM) or with fresh low-calcium media (50μM). Twenty-four hours later, cells were fixed and stained Phalloidin-iFluor 647 (Abcam, ab176759, 1:500) and DAPI.

### Quantification of mitotic stage and late mitotic cells with chromosomal segregation defects

Embryos were injected with lentiviruses encoding *shScr;H2B-GFP* or *shAnln;H2B-GFP* on E9 and harvested at E14.5. Whole-mount samples were immunolabeled for E-cadherin antibody (1:500) overnight at 4°C, followed by incubation with a secondary antibody and DAPI at room temperature for 2 h. Data were collected using a Nikon C2+ 60×/1.4 objective that generates optical sections of 0.49 μm at the middle of the basal layer. The mitotic stage of each cell was determined by chromosome organization (DAPI staining). In every microscopic field, the percentage of mitotic cells in each stage was calculated (number of mitotic cells in each stage/total number of mitotic cells). Every embryo was represented by a dorsal skin sample of ~0.5cm^2^. Defects in chromosomal segregation were determined by chromosome organization (H2B-GFP or DAPI staining).

### Quantification of F-actin intensity in vivo

Embryos were injected with lentiviruses encoding *shScr;H2B-GFP* or *shAnln;H2B-GFP* on E9 and harvested at E14.5, E15.5, and E16.5. Dorsal skin sections were incubated with Alexa Fluor 647-conjugated phalloidin overnight at 4°C followed by incubation with a secondary antibody and DAPI at room temperature for 2 h. Samples were imaged using confocal microscopy with a 60×/1.4 objective that generated optical sections of 0.49 μm. For F-actin quantification, basal and suprabasal layers were manually segmented based on staining with keratin 14.

Line scan analysis was performed using Fiji. Lines were drawn with the “freehand line tool” (ImageJ) with a width of 5 pixels, along the cortex of the cell or through the cell.

### Barrier assay

Epidermal barrier assay was performed as previously described [100]. Briefly, E18.5 embryos were collected and immersed in ice-cold methanol gradient in water, taking 2 min per step (1–25%, 2–50%, 3–75%, 4–100% methanol) and then rehydrated using the reverse procedure. Embryos were immersed in 0.2% toluidine blue solution. Embryos were washed in PBS before image capture.

### Jasplakinolide and calyculin A treatments

For in vivo jasplakinolide treatments, wild-type embryos were collected on E15.5 and incubated with 3μM Jasplakinolide (Sigma-Aldrich), 30 nM Calyculin A (Cell Signaling Technology), or DMSO in serum-free DMEM (Biological Industries) at 37°C for 2 h before embedding in OCT or processed for whole-mount preparation and immunofluorescence microscopy as described above. For in vitro treatments, keratinocytes were infected with *shScr;puromycin* or *shAnln;puromycin*, selected with 3 μg/ml puromycin (Sigma) and plated on fibronectin-coated coverslips (40,000 cells in a single well of a 24-well plate). Twenty-four hours later, the medium was switched to high calcium (1.5mM Ca^2+^) and cells were treated with 100nM jasplakinolide (Sigma-Aldrich) or 2nM calyculin A for 5 min, and then with 8μM nocodazole for 6 h. Cells were then fixed and labeled with Phalloidin-iFluor 647 (Abcam, ab176759) and pERM (Cell Signaling Technology, 1:200) overnight at 4°C. After washing, sections were incubated with a secondary antibody (1: 500 dilution) at room temperature for 1 h. Data was collected using a Nikon C2+ 60×/1.4 objective that generates optical sections of 0.49 μm at the middle of the cell.

### Live imaging of GFP-rGBD

Keratinocytes were infected with *shScr;puromycin* or *shAnln;puromycin* and selected with 3 μg/ml puromycin (Sigma). After selection, 10^5^ cells were plated in a single well of six-well plates, and GFP-rGBD was transfected with a PolyJet In Vitro DNA Transfection Reagent (SignaGen Laboratories), according to the manufacturer’s instructions. After 24 h, cells were plated in Ibidi μ-slide 8-well plates (Ibidi 111 GmbH, Germany) at a density of 30,000 cells/well. After 24 h, cells were switched to a high-calcium media (1.5mM Ca^2+^) supplemented with 8μM nocodazole (Sigma-Aldrich) for 6 h and imaged with a Nikon C2+ 100X/1.4 objective that generates optical sections of 0.49 μm at the middle of the cell. Line scan analysis was performed using Fiji. Lines were drawn with the “freehand line tool” with a width of 20 pixels, along the cortex of the cell. The percentage of positive cortical GFP-rGBD of mitotic cell was calculated as follows: (total cell edge length – negative GFP-rGBD along the cortex) × 100.

### Statistical analysis

Quantitative data are shown as the mean ± SD unless noted. Analyses were performed using Prism (GraphPad). Statistical tests of significance were determined by Student’s *t*-test (parametric), ANOVA followed by Tukey’s honest significant difference test (multiple groups), or Kolmogorov-Smirnov test (non-parametric) to evaluate cumulative frequency distributions. Sample sizes and the specific tests performed are indicated in the figure legends. No statistical method was used to predetermine the sample size. Experiments were not randomized, and investigators were not blinded to sample identity during experiments or outcome assessments.

## Supplementary Information

**Additional file 1: Figure S1.** ANLN-depleted epidermis exhibits defects in adherens junctions. Sagittal views of 10-μm sections of dorsal skin from control and *shAnln-926* KD E14.5, E15.5, and E16.5 embryos. Sections were immunostained for the adherens junction proteins α-catenin and β-catenin. Dotted lines indicate the dermal–epidermal border. Insets show the transduced cells (H2B−GFP+). Nuclei were stained with DAPI (blue). Scale bars = 20 μm.

**Additional file 2: Figure S2.** ANLN depletion leads to defects in integrin distribution. Sagittal views of 10-μm sections of dorsal skin from control (Ctrl) and *shAnln-926*-transduced E18.5 embryos immunolabeled for β4 integrin and total β1 integrin. Dotted lines indicate the dermal–epidermal border. Insets show the transduced cells (H2B-GFP+). Nuclei were stained with DAPI (blue). Scale bars = 20 μm.

**Additional file 3: Figure S3.** ANLN depletion by a second hairpin (*Anln*-2981) results in defects analogous to *Anln -926*. (A) Stereomicroscopic images of E16.5 embryos infected on E9 with *shAnln-2981;H2B-GFP* lentiviruses. (B-D) Sagittal views of 10-μm sections of dorsal skin from control and *shAnln*-*2981* KD E16.5 embryos. Sections were immunostained for the adherens junction proteins E-cadherin, a-catenin, and b-catenin (B), the basement membrane protein nidogen (C), and the polarity protein Par3 (D). Dotted lines indicate the dermal–epidermal border. Insets in B-D show the transduced cells (H2B−GFP+). Nuclei were stained with DAPI (blue). Scale bars = 20 μm.

**Additional file 4: Figure S4.** ANLN depletion induces hyperproliferation but does not hinder epidermal differentiation. (A) Sagittal views of 10-μm sections of dorsal skin from control (Ctrl) and *shAnln-926*-transduced E18.5 embryos immunolabeled to the cell proliferation marker Ki67, the basal layer Keratin 14, the spinous layer and differentiation marker Keratin 10, and the granular layer marker loricrin. (B) Sagittal views of 10-μm sections of dorsal skin from control and *Anln*-926, E16.5 embryos immunostained for the cleavage furrow marker survivin. Quantification of spindle orientation is presented below each image. (C) Toluidine blue barrier assay was performed on E18.5 *shAnln-926* transduced embryo and un-infected littermate (wild-type). Dotted lines indicate the dermal–epidermal border. Insets show the transduced cells (H2B-GFP+). Nuclei were stained with DAPI (blue). Scale bars = 20 μm.

**Additional file 5: Figure S5.** Normal mitotic spindle organization in ANLN-depleted epidermis. Whole-mount immunofluorescence of control and *Anln-926* KD E14.5 embryos immunostained for acetylated (Ac-) tubulin and pericentrin. Insets show the transduced cells (H2B−GFP+). Nuclei were stained with DAPI (blue). Scale bars = 20 μm.

**Additional file 6: Figure S6.** Monastrol treatment results in mitotic rounding defects in *Anln* KD cultured keratinocytes. (A) Wild-type keratinocytes were transduced with *shScr* (Ctrl) or *shAnln-926*, treated with monastrol for 6 hours, fixed, and labeled for F-actin. (B) Quantification of the early mitotic cell axial ratio from the data shown in A. Horizontal bars represent the mean, circles represent individual cells. n=118 and 144 Ctrl and *Anln-926* transduced cells, respectively, from three experiments. P<0.0001 by unpaired two-tailed t-test.

**Additional file 7: Figure S7.** ANLN depletion alters the distribution of cortical proteins in early mitotic cells. (A) Western blot analyses of primary mouse keratinocytes transduced with *shScr* (ctrl) or *Anln-926* shRNAs and probed with antibodies to β-actin, γ-actin, MYH9 (Myosin IIa heavy chain), pMLC, pERM, and GAPDH (loading control). (B) Same data as in (Fig. 7 D-G), plotted as a cumulative frequency distribution. P<0.0001 by a Kolmogorov-Smirnov test for F-actin, Myo-IIa, pMLC and pERM. (C) RhoA activity assays. Total protein extracts from *shScr* (Ctrl) or *Anln-926* transduced cells were probed with RhoA antibody or treated with GST-Rhotekin binding domain bound to glutathione-coupled sepharose beads to selectively pull down active GTP-RhoA.

**Additional file 8: **Uncropped blots.

## Data Availability

The datasets supporting the conclusions of this article are included within the article and its additional files.

## Abbreviations

BM: Basement membrane
F-actin: Filamentous actin
1^0^MKs: Primary mouse keratinocytes
Scr: Scramble
IF: Immunofluorescence
shRNAs: Short hairpin RNAs
H2B-GFP: GFP-tagged histone 2B reporter
KD: Knockdown
AJs: Adherens junctions
ERM: Ezrin, radixin, and moesin
pMLC: Phosphorylated myosin regulatory light chain
pERM: Phosphorylated ERM proteins
