# Supplementary figures and images for: Anillin governs mitotic rounding during early epidermal development
Source: BMC Biol. 2022 Jun 16;20:145. doi: 10.1186/s12915-022-01345-9 (PMC9205045; doi:10.1186/s12915-022-01345-9)

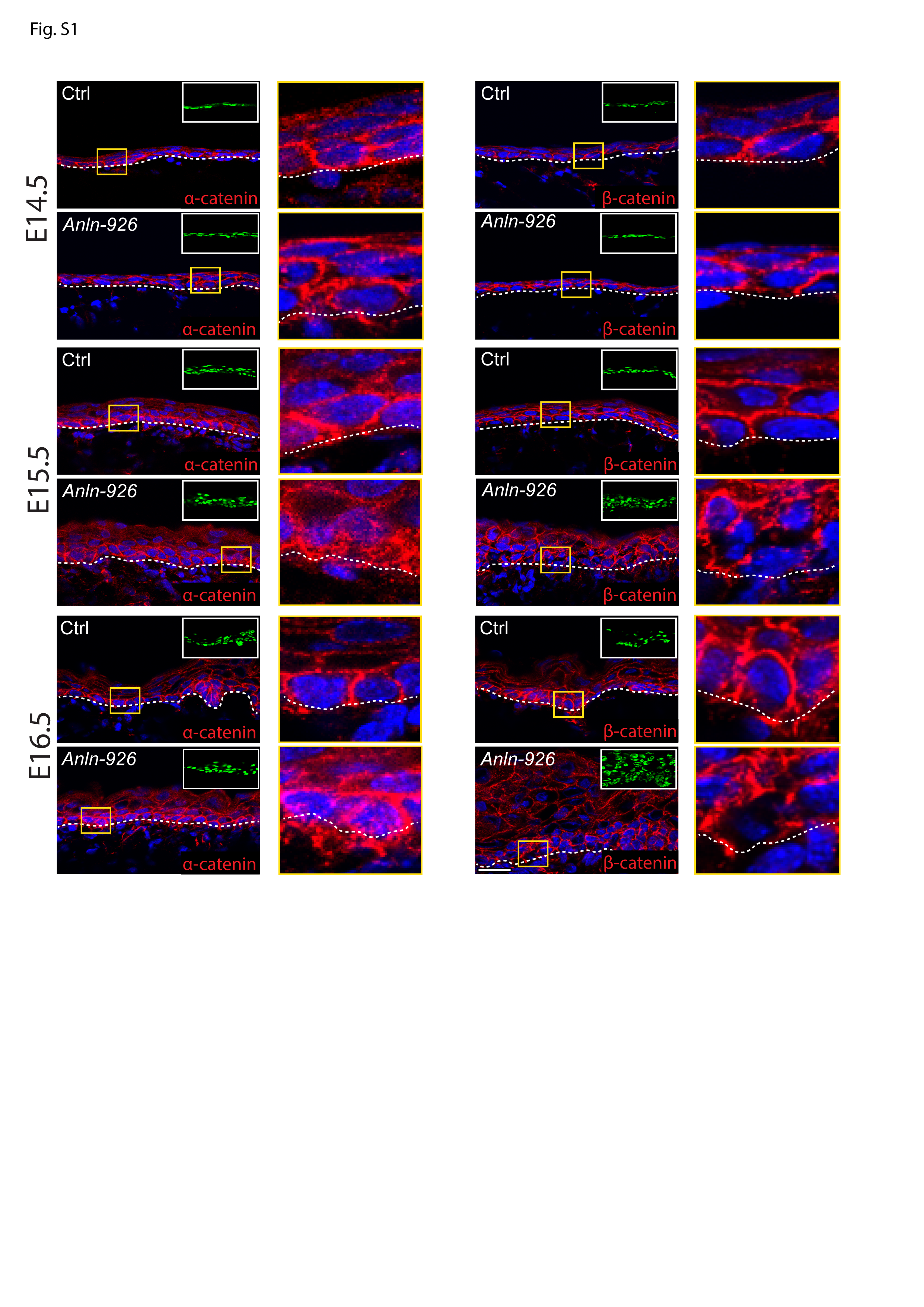

Supplement: Supplementary file 1 — Additional file 1: Figure S1. ANLN-depleted epidermis exhibits defects in adherens junctions. Sagittal views of 10-μm sections of dorsal skin from control and shAnln-926 KD E14.5, E15.5, and E16.5 embryos. Sections were immunostained for the adherens junction proteins α-catenin and β-catenin. Dotted lines indicate the dermal–epidermal border. Insets show the transduced cells (H2B−GFP+). Nuclei were stained with DAPI (blue). Scale bars = 20 μm. [file 12915_2022_1345_MOESM1_ESM.tif]

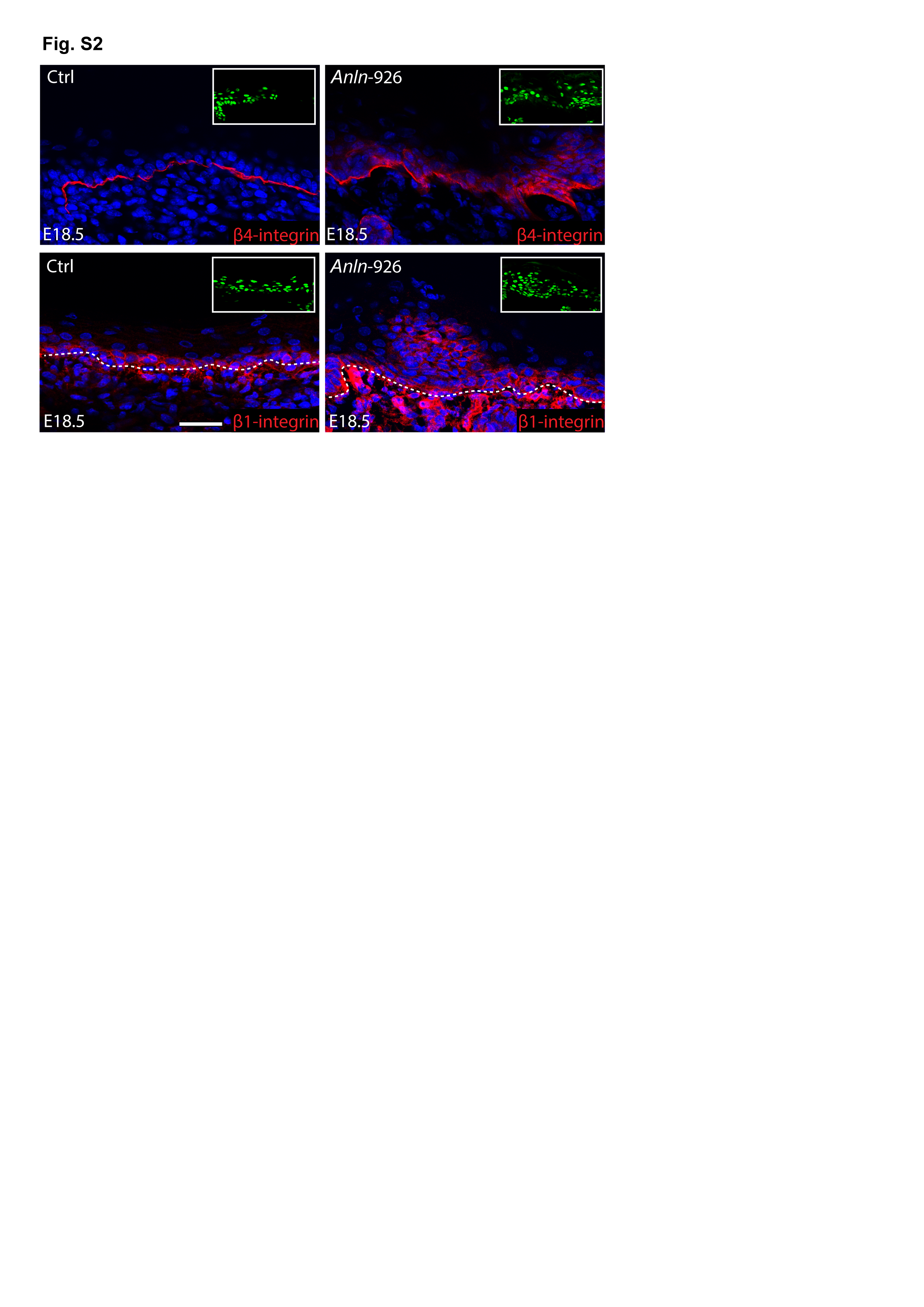

Supplement: Supplementary file 2 — Additional file 2: Figure S2. ANLN depletion leads to defects in integrin distribution. Sagittal views of 10-μm sections of dorsal skin from control (Ctrl) and shAnln-926-transduced E18.5 embryos immunolabeled for β4 integrin and total β1 integrin. Dotted lines indicate the dermal–epidermal border. Insets show the transduced cells (H2B-GFP+). Nuclei were stained with DAPI (blue). Scale bars = 20 μm. [file 12915_2022_1345_MOESM2_ESM.tif]

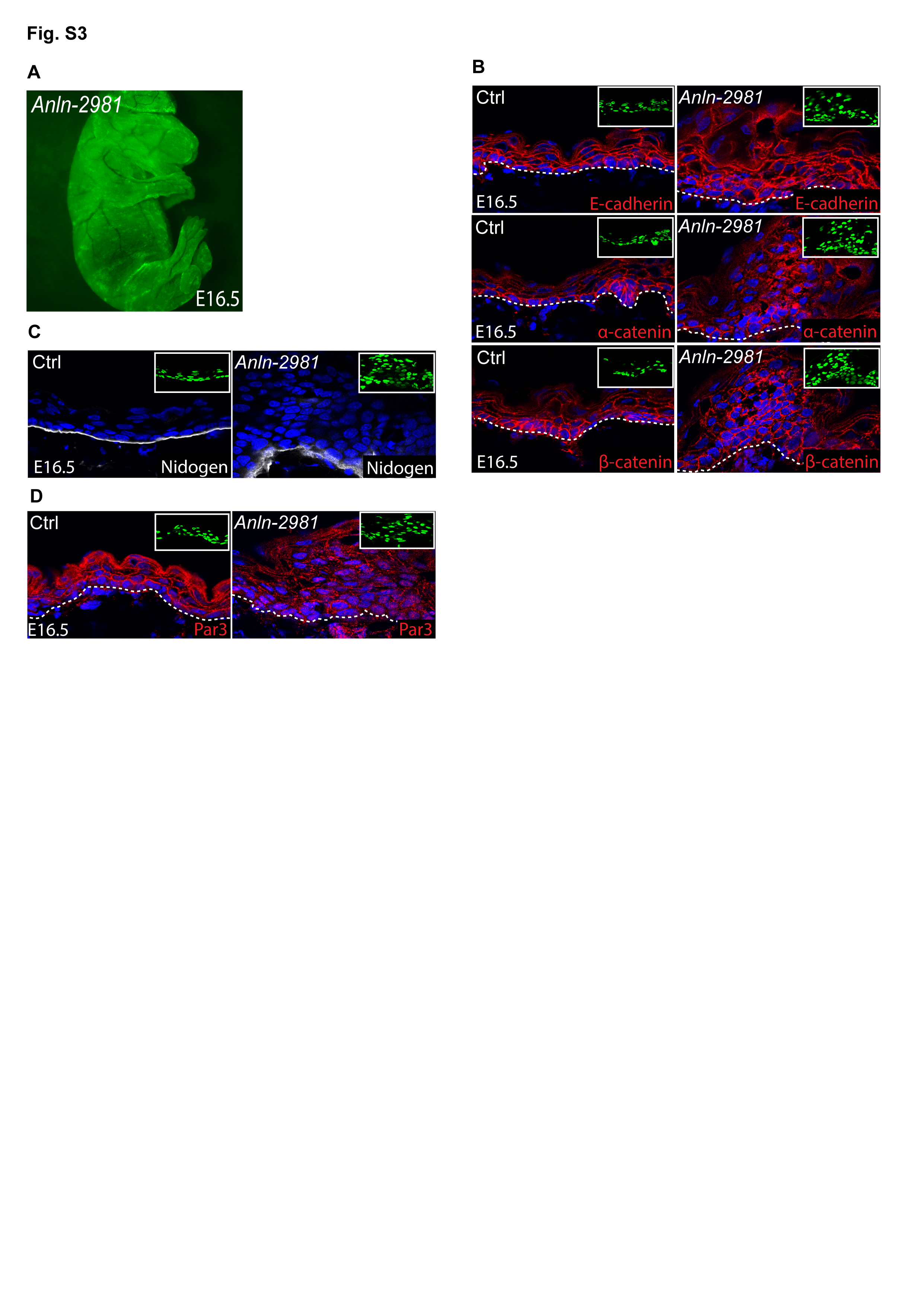

Supplement: Supplementary file 3 — Additional file 3: Figure S3. ANLN depletion by a second hairpin (Anln-2981) results in defects analogous to Anln -926. (A) Stereomicroscopic images of E16.5 embryos infected on E9 with shAnln-2981;H2B-GFP lentiviruses. (B-D) Sagittal views of 10-μm sections of dorsal skin from control and shAnln-2981 KD E16.5 embryos. Sections were immunostained for the adherens junction proteins E-cadherin, a-catenin, and b-catenin (B), the basement membrane protein nidogen (C), and the polarity protein Par3 (D). Dotted lines indicate the dermal–epidermal border. Insets in B-D show the transduced cells (H2B−GFP+). Nuclei were stained with DAPI (blue). Scale bars = 20 μm. [file 12915_2022_1345_MOESM3_ESM.tif]

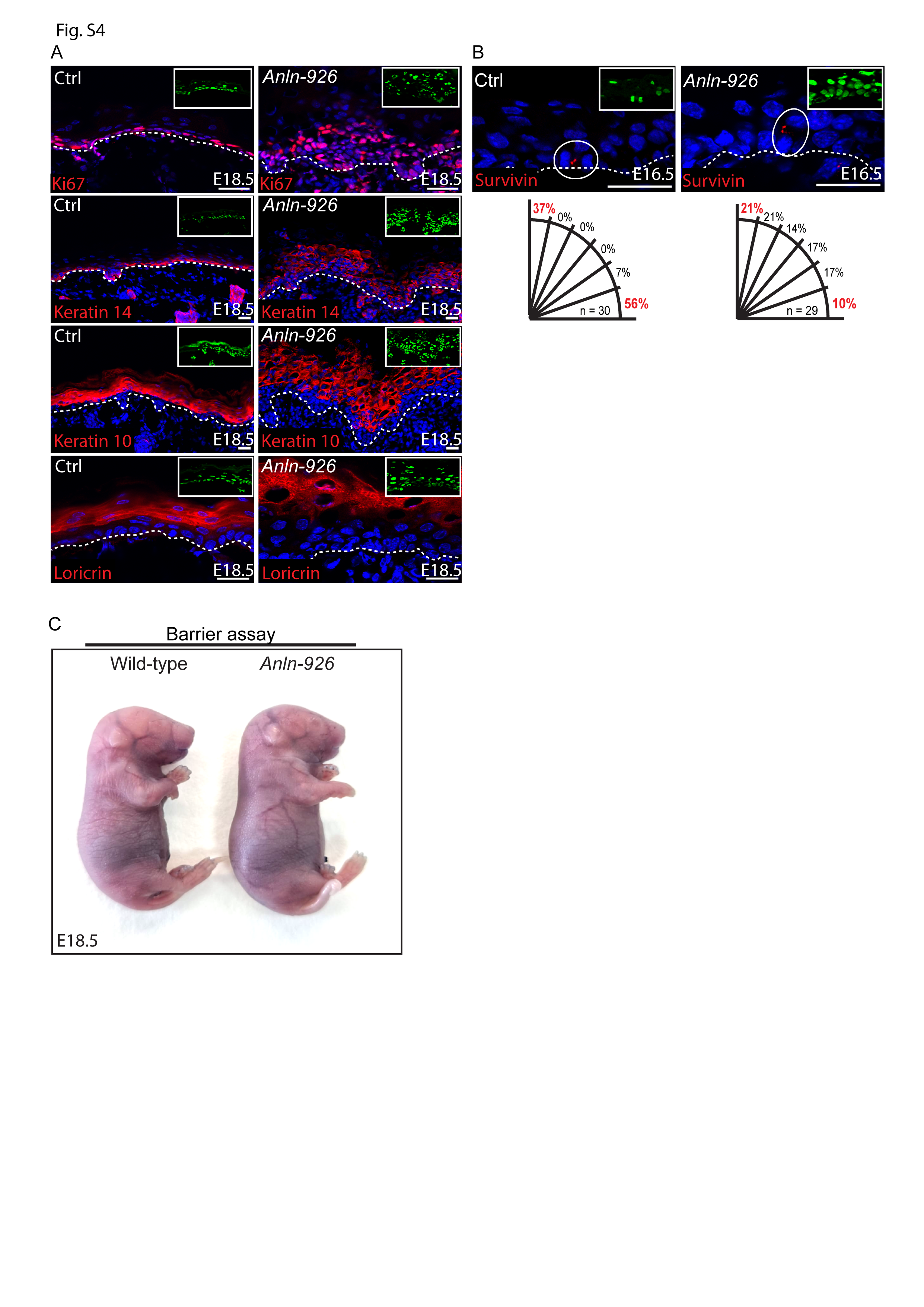

Supplement: Supplementary file 4 — Additional file 4: Figure S4. ANLN depletion induces hyperproliferation but does not hinder epidermal differentiation. (A) Sagittal views of 10-μm sections of dorsal skin from control (Ctrl) and shAnln-926-transduced E18.5 embryos immunolabeled to the cell proliferation marker Ki67, the basal layer Keratin 14, the spinous layer and differentiation marker Keratin 10, and the granular layer marker loricrin. (B) Sagittal views of 10-μm sections of dorsal skin from control and Anln-926, E16.5 embryos immunostained for the cleavage furrow marker survivin. Quantification of spindle orientation is presented below each image. (C) Toluidine blue barrier assay was performed on E18.5 shAnln-926 transduced embryo and un-infected littermate (wild-type). Dotted lines indicate the dermal–epidermal border. Insets show the transduced cells (H2B-GFP+). Nuclei were stained with DAPI (blue). Scale bars = 20 μm. [file 12915_2022_1345_MOESM4_ESM.tif]

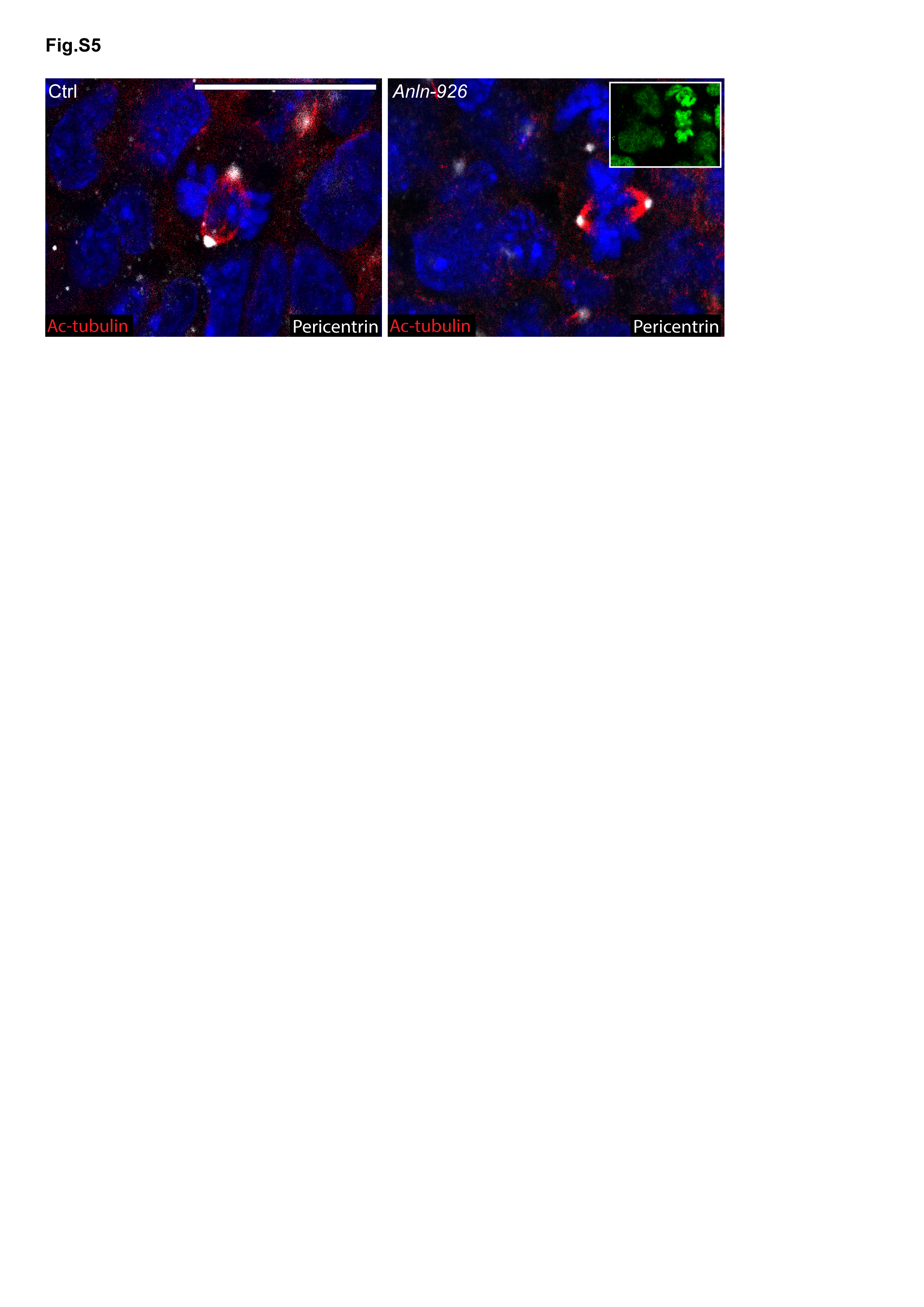

Supplement: Supplementary file 5 — Additional file 5: Figure S5. Normal mitotic spindle organization in ANLN-depleted epidermis. Whole-mount immunofluorescence of control and Anln-926 KD E14.5 embryos immunostained for acetylated (Ac-) tubulin and pericentrin. Insets show the transduced cells (H2B−GFP+). Nuclei were stained with DAPI (blue). Scale bars = 20 μm. [file 12915_2022_1345_MOESM5_ESM.tif]

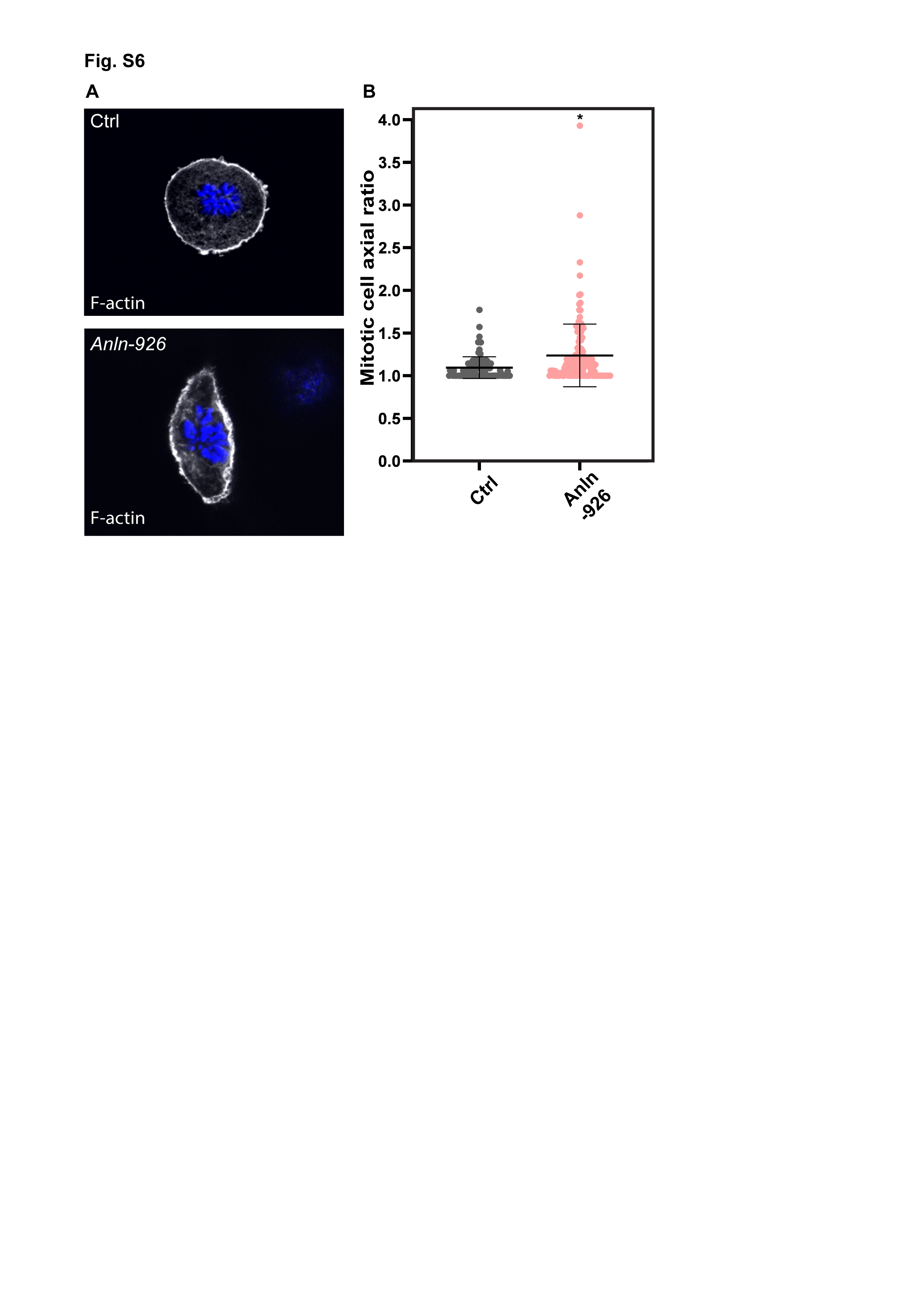

Supplement: Supplementary file 6 — Additional file 6: Figure S6. Monastrol treatment results in mitotic rounding defects in Anln KD cultured keratinocytes. (A) Wild-type keratinocytes were transduced with shScr (Ctrl) or shAnln-926, treated with monastrol for 6 hours, fixed, and labeled for F-actin. (B) Quantification of the early mitotic cell axial ratio from the data shown in A. Horizontal bars represent the mean, circles represent individual cells. n=118 and 144 Ctrl and Anln-926 transduced cells, respectively, from three experiments. P<0.0001 by unpaired two-tailed t-test. [file 12915_2022_1345_MOESM6_ESM.tif]

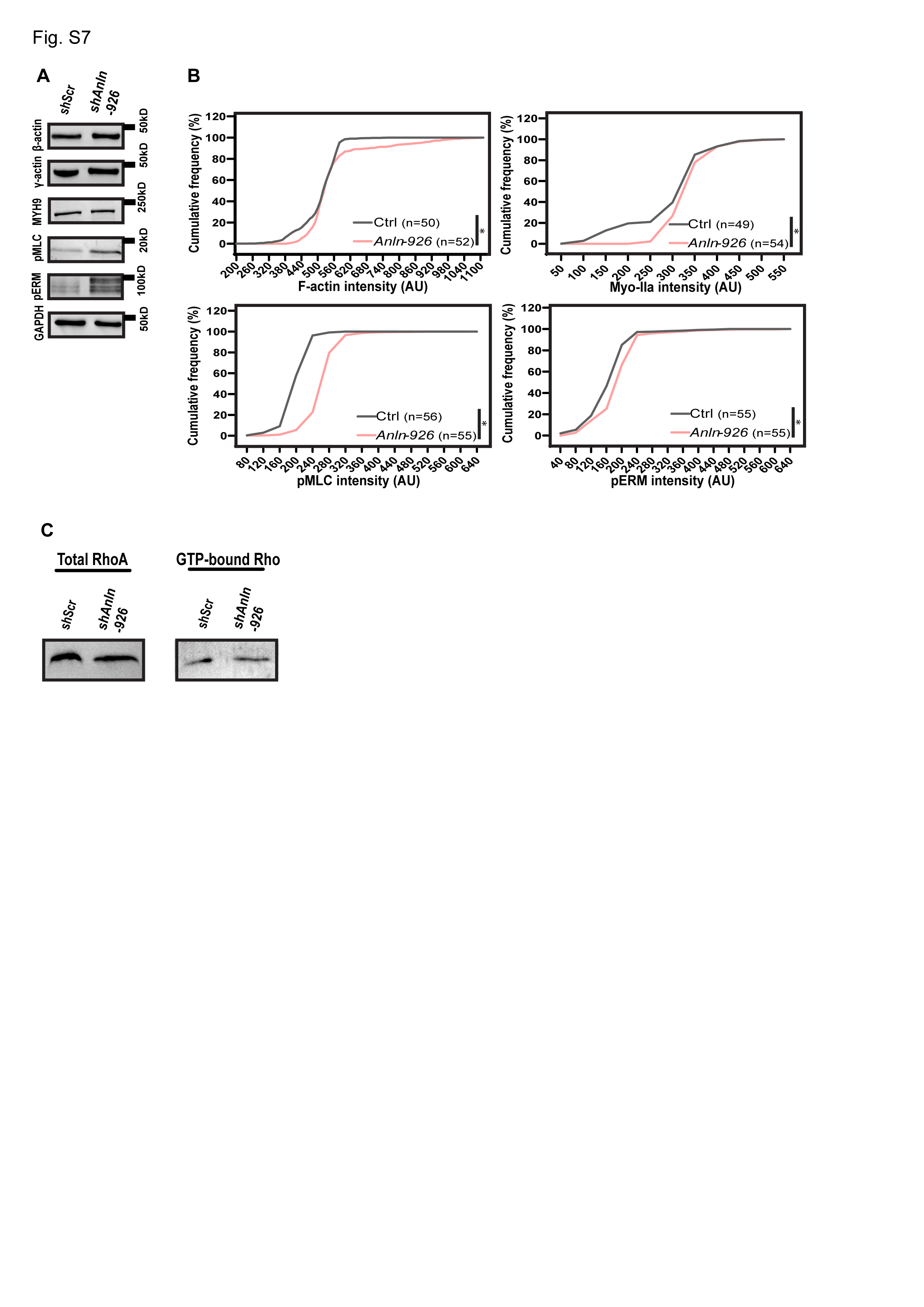

Supplement: Supplementary file 7 — Additional file 7: Figure S7. ANLN depletion alters the distribution of cortical proteins in early mitotic cells. (A) Western blot analyses of primary mouse keratinocytes transduced with shScr (ctrl) or Anln-926 shRNAs and probed with antibodies to β-actin, γ-actin, MYH9 (Myosin IIa heavy chain), pMLC, pERM, and GAPDH (loading control). (B) Same data as in (Fig. 7 D-G), plotted as a cumulative frequency distribution. P<0.0001 by a Kolmogorov-Smirnov test for F-actin, Myo-IIa, pMLC and pERM. (C) RhoA activity assays. Total protein extracts from shScr (Ctrl) or Anln-926 transduced cells were probed with RhoA antibody or treated with GST-Rhotekin binding domain bound to glutathione-coupled sepharose beads to selectively pull down active GTP-RhoA. [file 12915_2022_1345_MOESM7_ESM.tif]
