# Supplementary material for: Anillin governs mitotic rounding during early epidermal development
Source: BMC Biol. 2022 Jun 16;20:145. doi: 10.1186/s12915-022-01345-9 (PMC9205045; doi:10.1186/s12915-022-01345-9)
Supplement: Supplementary file 8 — Additional file 8: Uncropped blots. [file 12915_2022_1345_MOESM8_ESM.pdf]

Anillin governs mitotic rounding during early epidermal development

Mahly et al

This file contains uncropped western blots

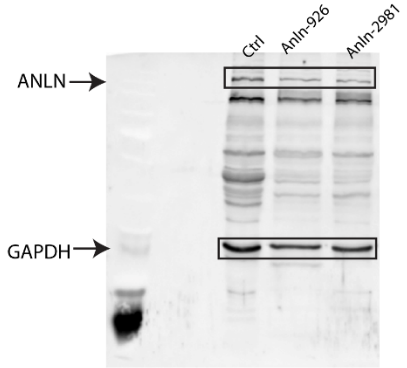

Fig. 1D) Western blot analyses of primary mouse keratinocytes transduced with Scr, Anln -926, or Anln -2981 shRNAs. Blots were probed with antibodies to ANLN and GAPDH (loading control).

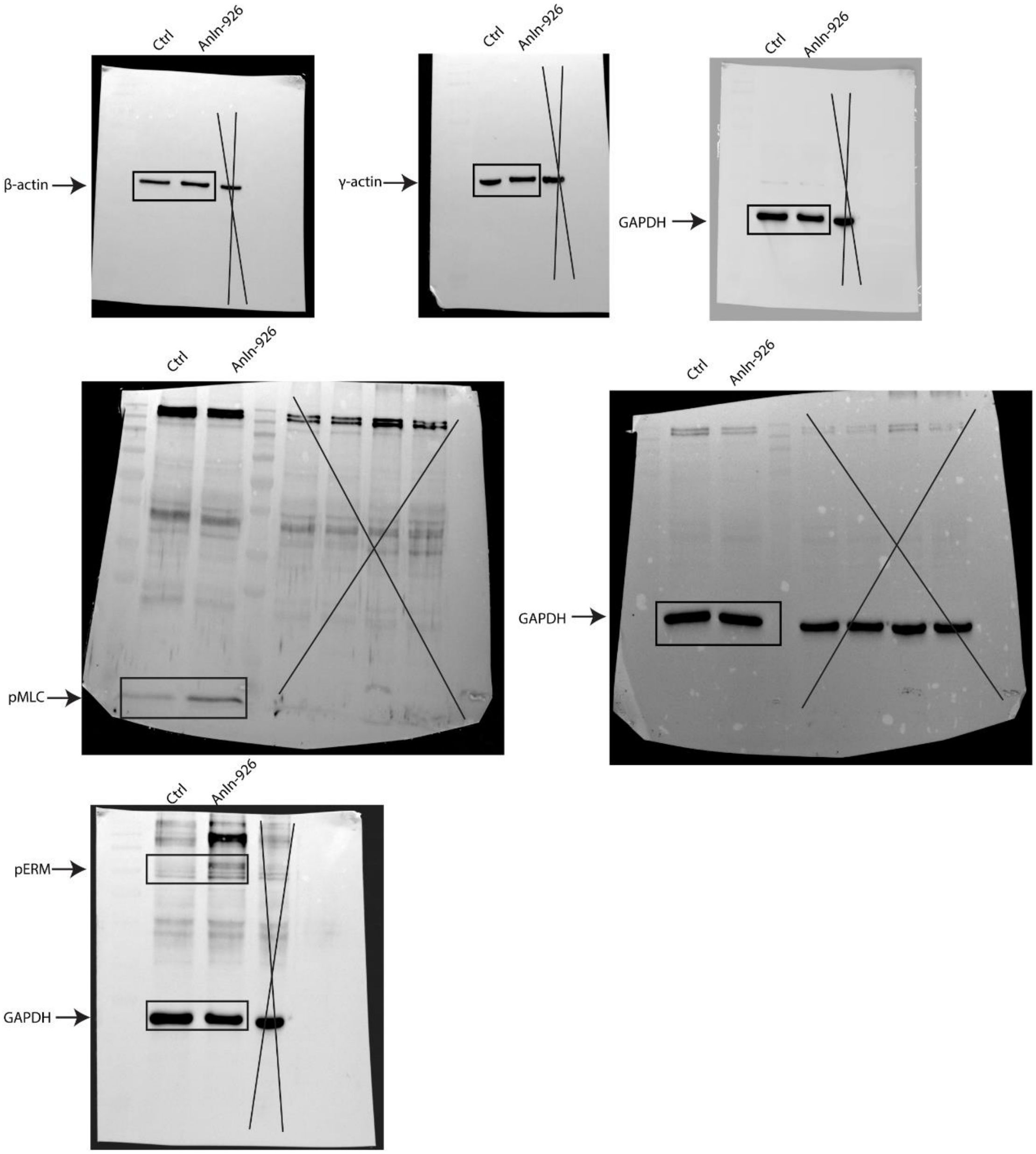

Fig. S7A) Western blot analyses of primary mouse keratinocytes transduced with shScr (ctrl) o r Anln-926 shRNAs and probed with antibodies to b-actin, g-actin, MYH9 (Myosin IIa heavy chain), pMLC, pERM, and GAPDH (loading control).

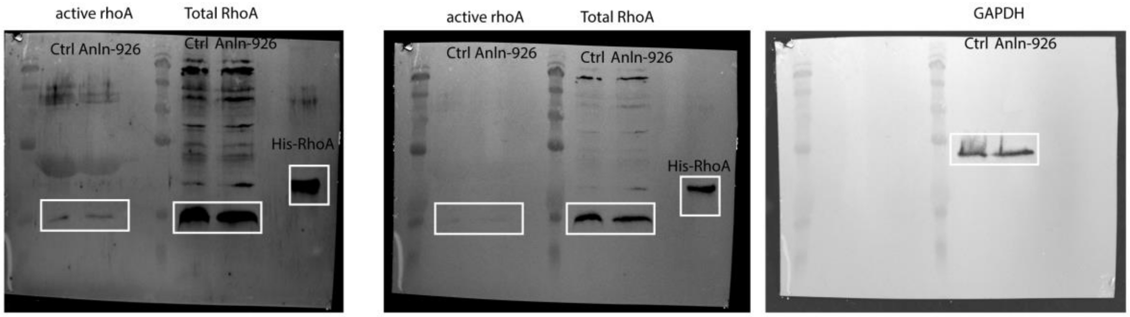

Fig. S7C) RhoA activity assays. Total protein extracts from shScr (Ctrl) or Anln-926 transduced cells were probed with RhoA antibody or treated with GST-Rhotekin binding domain bound to glutathione-coupled sepharose beads to selectively pull down active GTP-RhoA or with GAPDH (loading control).
